# Targeting FSCN1 with an oral small-molecule inhibitor for treating ocular neovascularization

**DOI:** 10.1186/s12967-023-04225-0

**Published:** 2023-08-18

**Authors:** Wen Bai, Jun-Song Ren, Min Xia, Ya Zhao, Jing-Juan Ding, Xi Chen, Qin Jiang

**Affiliations:** 1 The Affiliated Eye Hospital, Nanjing Medical University, Nanjing, China; 2 The Fourth School of Clinical Medicine, Nanjing Medical University, Nanjing, China; 3 Department of Ophthalmology, Northern Jiangsu People’s Hospital, Yangzhou, China

**Keywords:** Angiogenesis, FSCN1, NP-G2-044, Vascular tip cell, Ocular pathologies

## Abstract

**Background:**

Ocular neovascularization is a leading cause of blindness and visual impairment. While intravitreal anti-VEGF agents can be effective, they do have several drawbacks, such as endophthalmitis and drug resistance. Additional studies are necessary to explore alternative therapeutic targets.

**Methods:**

Bioinformatics analysis and quantitative RT-PCR were used to detect and verify the FSCN1 expression levels in oxygen-induced retinopathy (OIR) and laser-induced choroidal neovascularization (CNV) mice model. Transwell, wound scratching, tube formation, three-dimensional bead sprouting assay, rhodamine-phalloidin staining, Isolectin B4 staining and immunofluorescent staining were conducted to detect the role of FSCN1 and its oral inhibitor NP-G2-044 in vivo and vitro. HPLC–MS/MS analysis, cell apoptosis assay, MTT assay, H&E and tunnel staining, visual electrophysiology testing, visual cliff test and light/dark transition test were conducted to assess the pharmacokinetic and security of NP-G2-044 in vivo and vitro. Co-Immunoprecipitation, qRT-PCR and western blot were conducted to reveal the mechanism of FSCN1 and NP-G2-044 mediated pathological ocular neovascularization.

**Results:**

We discovered that Fascin homologue 1 (FSCN1) is vital for angiogenesis both in vitro and in vivo, and that it is highly expressed in oxygen-induced retinopathy (OIR) and laser-induced choroidal neovascularization (CNV). We found that NP-G2-044, a small-molecule inhibitor of FSCN1 with oral activity, can impede the sprouting, migration, and filopodia formation of cultured endothelial cells. Oral NP-G2-044 can effectively and safely curb the development of OIR and CNV, and increase efficacy while overcoming anti-VEGF resistance in combination with intravitreal aflibercept (Eylea) injection.

**Conclusion:**

Collectively, FSCN1 inhibition could serve as a promising therapeutic approach to block ocular neovascularization.

**Supplementary Information:**

The online version contains supplementary material available at 10.1186/s12967-023-04225-0.

## Introduction

Ocular neovascularization is a common pathological feature associated with multiple ocular diseases, occurring mainly in various ocular structures such as the cornea, iris, retina, and choroid [1]. If left undertreated, it could ultimately lead to permanent blindness due to the leakage of vascular fluid. Strategies targeting pathological angiogenesis have primarily focused on blocking vascular endothelial growth factor A (VEGF), a major driving factor of the angiogenic process [2, 3]. Currently, the mainstay treatment paradigm for the management of pathological ocular neovascularization is intravitreal anti-VEGF therapy (ranibizumab, bevacizumab, and aflibercept) [4]. However, the efficacy of monotherapy is limited by various factors such as resistance, short duration, repetitive injections, adverse effects, etc.[5, 6]. Therefore, there is a great unmet medical need for alternative or additional therapies to reduce the treatment burden on patients, especially anti-VEGF non-responders. It is essential to find new targets for treating pathological ocular neovascularization through the VEGF-independent angiogenesis pathway.

Pathological neovascularization is a complex and precise process that relies on the coordinated actions of three specialized endothelial cell types: motile tip cells, proliferating stalk cells, and quiescent phalanx cells [7, 8]. Recent evidence suggests that tip cells play a critical role in initiating aberrant angiogenesis [9–11]. These cells are located at the distal end of angiogenic sprouts and act as pioneers, directing angiogenic direction through filopodia extensions that sense VEGF gradients [12]. Tip cells express a high level of vascular endothelial growth factor receptor 2 (VEGFR2) to sense VEGF signaling [9, 13], as well as other specific molecular signatures such as DLL4 [14], APLN [11], CXCR4 [15], ESM1 [16], to prepare for tip cell formation or assist tip cell migration. Despite these findings, the molecular mechanisms that control tip cell behaviors remain largely unknown. Given the unique functional and molecular signatures of tip cells, targeting them has been proposed as a potential therapeutic strategy for pathological ocular neovascularization [17, 18]. Molecules involved in tip cell behaviors could therefore serve as promising drug targets for novel anti-angiogenic therapies that complement current treatment regimens.

Fascin homologue 1 (FSCN1), a member of the Fascin protein family of actin-binding proteins, has been identified as an evolutionarily conserved protein that crosslinks filamentous actin (F-actin) into tight bundles through its actin-binding domain (ABD) [19, 20]. In mammals, the Fascin protein family comprises three members, with FSCN2 and FSCN3 predominantly expressed in retina photoreceptors and the testis, respectively [21, 22]. During embryogenesis, FSCN1 is broadly expressed in developing neural systems and mesenchymal tissues[23], while in adults, it is present at low levels in dendritic cells, neuronal cells, glial cells, and some vascular endothelial cells [20, 24]. However, aberrantly high expression of FSCN1 in the majority of cancers has made it a growing focus of attention in the cancer field, with a dramatic rise in research publications over the last decade [25–29]. Recent reviews have summarized the state-of-the-art oncology research of FSCN1, focusing on its function, mechanism, and clinical significance [30–32]. FSCN1 has established roles in cell migration[19, 26], adhesion[33], and protrusion (filopodia and lamellipodia) [34, 35], cytoskeleton remodeling[32], mesenchymal transition[36], glycolysis[36], ferroptosis[28], and is associated with carcinoma invasion and metastasis[29, 37].

While FSCN1 is known to play an important role in filopodia outgrowth [34], little is known about its potential functions and molecular mechanisms in vascular endothelial cells, particularly tip cells. However, RNA-seq data evidence provided by Ma et al. suggests that FSCN1 is upregulated in ECs during normal sprouting [38]. Characterization of FSCN1 knockout mice has shown that FSCN1 is dispensable for brain development [23], but indispensable for developmental retina angiogenesis and tumor angiogenesis [38]. However, no studies have analyzed the impact of FSCN1 on pathological ocular neovascularization. Given expression pattern of FSCN1 in the whole body and its ability to target vascular endothelial cells, it is a promising candidate for the treatment of pathological ocular neovascularization.

NP-G2-044, developed by Han et al. in China [39], is a fat-soluble, orally active small-molecule inhibitor that targets FSCN1 by binding to its actin-binding domain (ABD) [40, 41]. By effectively blocking F-actin cross-linking activity of FSCN1[40], NP-G2-044 has demonstrated anti-tumor metastasis effects in preclinical studies[28, 42–46] and in a phase Ia clinical trial for patients with metastatic or advanced refractory solid tumor malignancies (Clinicaltrials.gov NCT03199586). Currently, a phase I/II clinical trial is being prepared to investigate the synergistic effects of combined NP-G2-044 with anti-PD-(L)1 therapy (Clinicaltrials.gov NCT05023486). The preclinical and clinical pharmacokinetic properties of NP-G2-044 are favorable, with good oral bioavailability in plasma and a long half-life in humans [44]. Moreover, NP-G2-044 has shown an excellent safety profile, with no dose-limiting toxicities (DLTs), drug-related serious adverse events (SAEs), or patient deaths observed (Clinicaltrials.gov NCT03199586). Based on these promising results, the oral option of NP-G2-044 has the potential to provide another valuable treatment option for pathological ocular neovascularization and could serve as an attractive alternative to intravitreally injected anti-VEGF drugs.

This study reveals that FSCN1 acts as a regulator of YAP nucleocytoplasmic shuttling at tip cells and plays a key role in sprouting angiogenesis by governing tip cell behaviors. We provide solid evidence that NP-G2-044 is an established, safe, and permeable orally anti-angiogenic drug for pathological ocular neovascularization. Furthermore, we comparatively assess the effects of NP-G2-044 monotherapy, anti-VEGF monotherapy, and anti-VEGF/NP-G2-044 combined therapy on choroidal neovascularization, suggesting that the combination of anti-VEGF and NP-G2-044 treatment not only increases the efficacy of anti-VEGF therapy but also effectively overcomes anti-VEGF resistance.

## Results

### FSCN1 expression is upregulated and co-localized with pathological retinal neovascular tufts

Several in vivo models have been developed to study mechanisms of pathological ocular neovascularization, such as the oxygen-induced retinopathy (OIR) model [47] and the laser-induced choroidal neovascularization (CNV) model [48]. These models provide suitable platforms to investigate the mechanisms of pathological angiogenesis and screen novel anti-angiogenic agents.

**Fig. 1:**
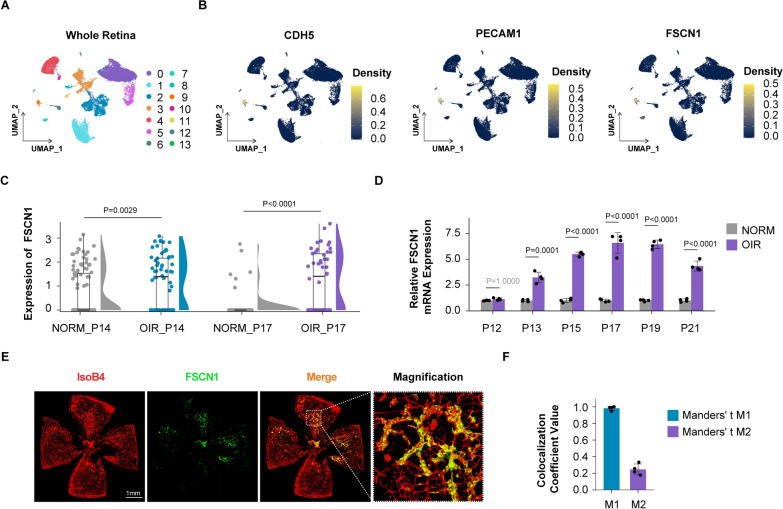
FSCN1 is highly expressed and colocalized in pathological retinal neovascularization** A** Clustering is performed using resolution = 0.2 and visualized using UMAP algorithm in the publicly available scRNA-seq data (GEO: #GSE150703) of OIR mice through the Seruat package. **B** Cross validation of the identified EC marker genes (CDH5 and PECAM1) and FSCN1 in the publicly available scRNA-seq data (GEO: #GSE150703) of OIR mice by density plot through the Nebulosa package. The right side of the plot shows the scale of expression density. **C** Violin and scatter plots show the expression of FSCN1 from NORM_P14, OIR_P14, NORM_P17 and OIR_P17 groups in the publicly available scRNA-seq data (GEO: #GSE150703) of OIR mice. Each dot indicates an endothelial cell. Results are presented as mean ± SEM, statistical analyses were performed using Kruskal–Wallis with Bonferroni's post hoc test. **P = 0.0029 (OIR_P14 vs NORM_P14); ***P < 0.0001 (OIR_P17 vs NORM_P17). **D** Quantitative reverse‐transcription polymerase chain reactions (qRT–PCRs) are performed to detect FSCN1 levels in the retinal ECs of OIR mice at P12, P13, P15, P17, P19 and P21. Results are presented as mean ± SEM, statistical analyses were performed using One-way ANOVA with Bonferroni's post hoc test. (n = 4 mice per group for each time point tested, data pooled from 4 independent experiments). **E**, **F** Colocalization of FSCN1 and pathological retinal neovascularization (IsoB4: red; FSCN1: green). Scale bar:1 mm. Quantification of Manders' coefficient (right panel). Results are presented as mean ± SEM. (n = 4 independent experiments). (M1: red pixels overlapping green pixels, M2: green pixels overlapping red pixels)

Firstly, we re-analyzed publicly available scRNA-seq data (GEO: #GSE150703 [49]) of oxygen-induced retinopathy mice (OIR) and normoxic mice (NORM) to investigate the cellular localization of FSCN1 in the retina. Single cells of the whole retina were classified into 14 clusters using the non-linear dimensionality reduction method UMAP (Fig. 1A). UMAP projection clustering and cell marker analysis allowed us to identify specific cell populations. Based on UMAP projection, we developed an expression density plot that shows cluster 9, consistently labeled by the EC marker genes (CDH5 and PECAM1), was identified as retinal EC populations (Fig. 1B). A specific high expression density of FSCN1 was detected on EC populations (Fig. 1B).

Next, we extracted the scRNA-seq data for retinal EC populations to compare the normalized expression level of FSCN1 between OIR and NORM groups (Fig. 1C), and the result was verified by qRT-PCR (Fig. 1D). Both bioinformatics analysis and qRT-PCR assay showed that FSCN1 mRNA expression was upregulated in retinal ECs of OIR mice and reached a peak on postnatal day 17 (P17) when the peak of the retinal neovascularization is reached. Immunofluorescence colocalization analysis of retina slice in OIR model was consistent with the bioinformatics analysis and showed that FSCN1 was co-localized with retinal vasculature, especially pathological retinal neovascular tufts (Fig. 1E, F).

### FSCN1 expression is elevated and co-localized with pathological choroidal neovascularization (CNV)

**Fig. 2:**
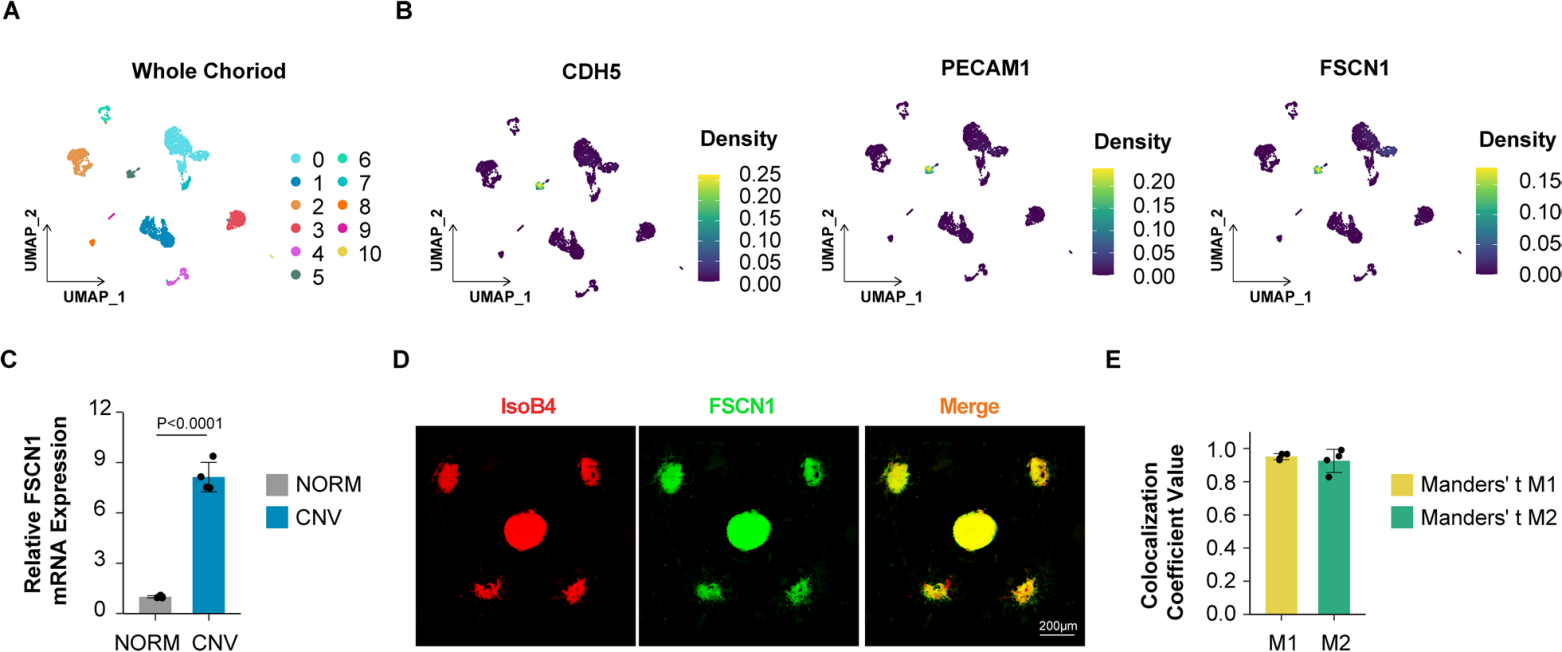
FSCN1 is highly expressed and colocalized in pathological choroidal neovascularization** A** Clustering is performed using resolution = 0.1 and visualized using UMAP algorithm from the publicly available scRNA-seq data of AMD patients (GEO: #GSE135922) through the Seruat package. **B** Cross validation of the identified EC marker genes (CDH5 and PECAM1) and FSCN1 from the publicly available scRNA-seq data (GEO: #GSE135922) of AMD patients by density plot through the Nebulosa package. The right side of the plot shows the scale of expression density. **C** Quantitative reverse‐transcription polymerase chain reactions (qRT–PCRs) were performed to detect FSCN1 levels in primary choroidal endothelial cells of mice at 7 days after laser-induced choroidal neovascularization (CNV). Results are presented as mean ± SEM, statistical analyses were performed using two-tailed student's t-test. (n = 4 mice per group, data pooled from 4 independent experiments). **D, E** Colocalization of FSCN1 and pathological choroidal neovascularization (IsoB4: red; FSCN1: green). Scale bar: 200 μm. Quantification of Manders' coefficient (right panel). Results are presented as mean ± SEM. (n = 4 independent experiments). (M1: red pixels overlapping green pixels, M2: green pixels overlapping red pixels)

To explore the cellular distribution of FSCN1 in the choroid, publicly available single-cell RNA sequencing (scRNA-seq) data (GEO: #GSE135922 [50] of choroidal tissues from donors with wet age-related macular degeneration (wAMD) and donors without AMD (NORM) were re-analyzed. Clustering analysis of the entire choroids identified 11 clusters in a two-dimensional UMAP (Fig. 2A). Among these clusters, Cluster 5 not only displayed endothelial features of CDH5 and PECAM1 expression, but also exhibited unique features of FSCN1 expression, indicating that FSCN1 was specifically expressed in choroidal endothelial cell populations (Fig. 2B). The expression of FSCN1 in primary choroidal endothelial cells from laser-induced choroidal neovascularization (CNV) mice and age/sex-matched control (WT) mice was determined using a qRT-PCR assay, which revealed a ~ 8.15-fold upregulation of FSCN1 mRNA levels in the CNV group compared to the WT group (Fig. 2C). Consistent with these findings, immunofluorescence colocalization analysis of choroid flatmounts in the CNV model showed that FSCN1 was co-localized with pathological choroidal neovascularization (Fig. 2D, E ).

### FSCN1 affects the angiogenic function of vascular endothelial cells in vitro

**Fig. 3:**
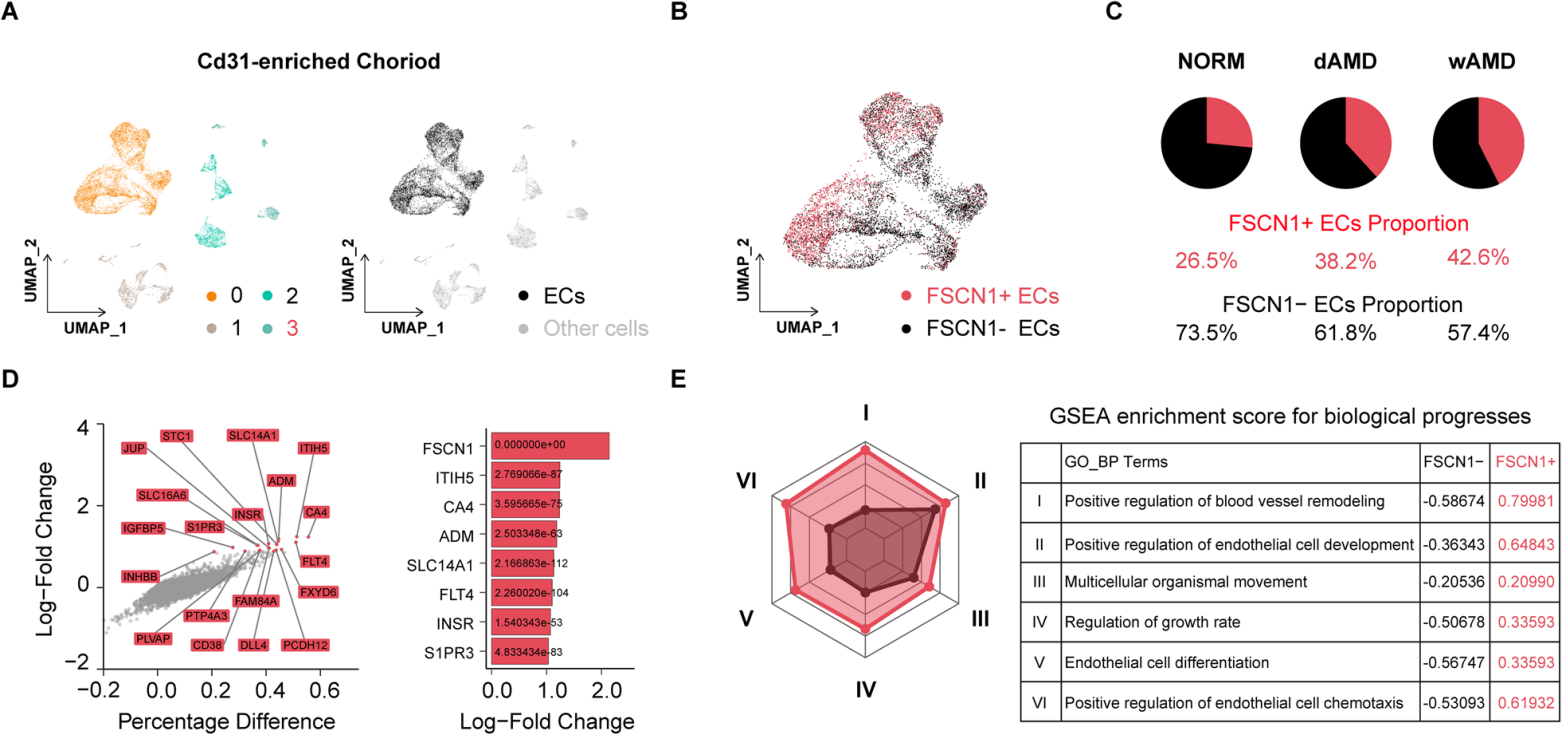
Functional annotations for differentially expressed genes (DEGs) between FSCN1 + ECs and FSCN1- ECs.** A** Clustering is performed in the left panel using resolution = 0.1 and visualized using UMAP algorithm from the publicly CD31-enriched scRNA-seq data of AMD patients (GEO: #GSE135922) through the Seruat package. The endothelial cells (cluster 0) are labeled (black dots) on the UMAP plot in the left panel. **B** FSCN1 + ECs (red dots) and FSCN1- ECs (black dots) are shown on the UMAP plot. **C** Proportional distribution of FSCN1 + ECs (red sector) and FSCN1- ECs (black sector) in wAMD, dAMD, NORM donors is represented by pie charts. **D** DEGs are displayed by using the log-fold change expression and the difference in the percentage of cells expressing the gene comparing FSCN1 + ECs versus FSCN1- ECs (Percentage Difference). In its left panel, genes labeled are top 20 genes sorted by log-fold change. In its right panel, the top 8 genes are displayed with adjusted P-value < 0.05.**E** The normalized enrichment scores (NES) of angiogenesis related biological progresses (BP) through GSEA analysis are calculated. In its left panel, Radar chart of NES is plotted in the FSCN1 + ECs (red area) versus FSCN1- ECs (black area). In its right panel, detailed NES values are displayed

Considering specifically vascular endothelial localization of FSCN1 in retina and choroid, CD31-enriched scRNA-seq data (GEO: #GSE135922 [50]) of choroidal tissues from donors with wet age-related macular degeneration (wAMD), donors with dry age-related macular degeneration (dAMD) and donors without AMD (NORM) were re-analyzed with higher endothelial cell abundance [50]. After filtering, 13,752 cells were retained, and 8,328 (60.56%) were identified as endothelial cells (cluster 0) (Fig. 3A). Among the endothelial cells, 3,908 expressed FSCN1 (FSCN1 + ECs), and 4,420 did not express FSCN1 (FSCN1- ECs) (Fig. 3B). Interestingly, FSCN1 + ECs were most abundant in donors with wAMD (Fig. 3C).

Given the challenge of understanding the role of genes in disease, the study aimed to identify differentially expressed genes (DEGs) between FSCN1 + and FSCN1- ECs (Fig. 3D). The top 8 up-regulated DEGs were FSCN1, ITIH5, CA4, ADM, SLC14A1, FLT4, INSR, and SIPR3 (Fig. 3D). To gain insight into the biological function of these DEGs, the researchers performed gene set enrichment analysis (GSEA) and found that six biological processes were significantly enriched with DEGs, which displayed a strong association with the angiogenesis phenotype (Fig. 3E). These processes included positive regulation of blood vessel remodeling, positive regulation of endothelial cell development, multicellular organismal movement, regulation of growth rate, endothelial cell differentiation, and positive regulation of endothelial cell chemotaxis.

**Fig. 4:**
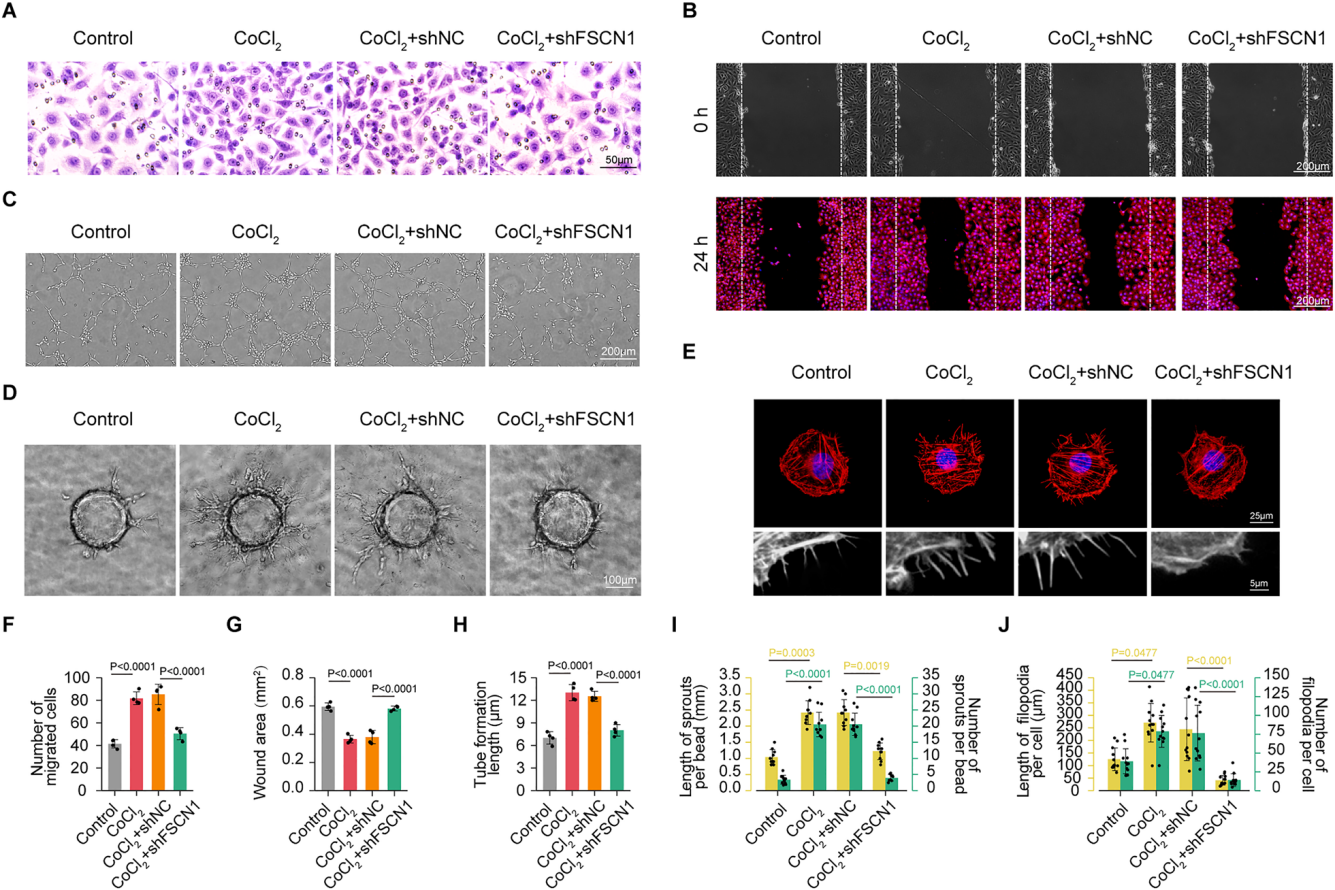
FSCN1 shRNA could reverse the upregulation of tube formation, migration and sprouting capabilities of HRMECs under hypoxia. HRMECs were transfected with non-specific sequences shRNA (shNC), FSCN1 shRNA, or left untreated and then exposed to CoCl_2_ (200 μmol/L) for 24 h. The group without any treatment is taken as the control group. **A** Transwell assays of HRMECs under hypoxia factors (CoCl_2_) stimulation or simultaneously transfected with shFSCN1. Scale bar: 50 µm. (n = 4 per group, data pooled from 4 independent experiments). **B** Wound scratching assays of HRMECs under hypoxia factors (CoCl_2_) stimulation or simultaneously transfected with shFSCN1. Scale bar: 200 µm. [rhodamine-conjugated phalloidin: red (to visualize the actin cytoskeleton); DAPI: blue (to visualize nuclei)]. (n = 4 per group, data pooled from 4 independent experiments). **C** Tube formation Assay of HRMECs under hypoxia factors (CoCl_2_) stimulation or simultaneously transfected with shFSCN1. Scale bar: 200 µm. (n = 4 per group, data pooled from 4 independent experiments). **D** Three-dimensional (3D) Bead Sprouting Assay reveals the in vitro sprouting capabilities of HRMECs under hypoxia factors (CoCl_2_) stimulation or simultaneously transfected with shFSCN1. Scale bar: 100 µm. (n = 10 per group, data pooled from 4 independent experiments). **E** Rhodamine-phalloidin staining (top panels) reveals the actin cytoskeleton and filopodia of HRMECs under hypoxia factors (CoCl_2_) stimulation or simultaneously transfected with shFSCN1 (rhodamine-conjugated phalloidin: red; DAPI: blue). Scale bar: 25 µm. The down panels represent a partial magnification to see filamentous pseudopodia in more detail. (n = 12 per group, data pooled from 4 independent experiments). **F** Quantification of migrated cells. Results are presented as mean ± SEM, statistical analyses were performed using One-way ANOVA with Bonferroni's post hoc test. (n = 4 per group, data pooled from 4 independent experiments). **G** Quantification of wound area. The top panels represent the initial state of the wound area at 0 h, and the down panels represent the area after a period of 24 h of hypoxic migration. Results are presented as mean ± SEM, statistical analyses were performed using One-way ANOVA with Bonferroni's post hoc test. (n = 4 per group, data pooled from 4 independent experiments). **H** Quantification of Tube formation length. Results are presented as mean ± SEM, statistical analyses were performed using One-way ANOVA with Bonferroni's post hoc test. (n = 4 per group, data pooled from 4 independent experiments). **I** Quantification of length and number of sprouts per bead. Results are presented as mean ± SEM, statistical analyses were performed using Kruskal–Wallis with Bonferroni's post hoc test. (n = 10 per group, data pooled from 4 independent experiments). **J** Quantification of length and number of filopodia per cell. Results are presented as mean ± SEM, statistical analyses were performed using Kruskal–Wallis with Bonferroni's post hoc test. (n = 12 per group, data pooled from 4 independent experiments)

To verify the function of FSCN1 in vitro, the researchers stably transfected HRMECs with FSCN1 knockdown lentivirus (shFSCN1) or a control knockdown lentivirus carrying non-specific sequences (shNC). After growth selection with kanamycin, the knockdown efficiency was detected by western blot (~ 88% knockdown efficiency) (Additional file 1: Figure S1A). The researchers performed a tube formation assay, transwell assay, wound scratching assay, and three-dimensional (3D) bead sprouting assay after hypoxia exposure in HRMECs (simulated by 200 µM CoCl_2_ for 24 h). Hypoxia exposure significantly increased tube formation, migration, and sprouting capabilities of HRMECs, while these effects could be reversed by FSCN1 knockdown (Fig. 4A–D, F–I). Vascular endothelial cells require morphologic changes in a directed fashion, such as reorganization of the actin cytoskeleton and the concomitant extension of cellular protrusions, including filopodia and lamellipodia, to undergo directed migration and sprouting. To visualize the actin cytoskeleton and filopodia, Rhodamine-phalloidin staining was performed after hypoxia exposure in HRMECs (simulated by 200 µM CoCl_2_ for 24 h). The researchers observed that FSCN1 knockdown reversed the hypoxia-induced morphologic changes of HRMECs, affecting the cytoskeleton structure with the loss of filopodia (Fig. 4E–J). In conclusion, the results suggest that FSCN1 can promote angiogenesis in vitro by reorganizing actin filaments and increasing cell motility.

### FSCN1, localized to vascular tip cells, governs tip cell behaviors

**Fig. 5:**
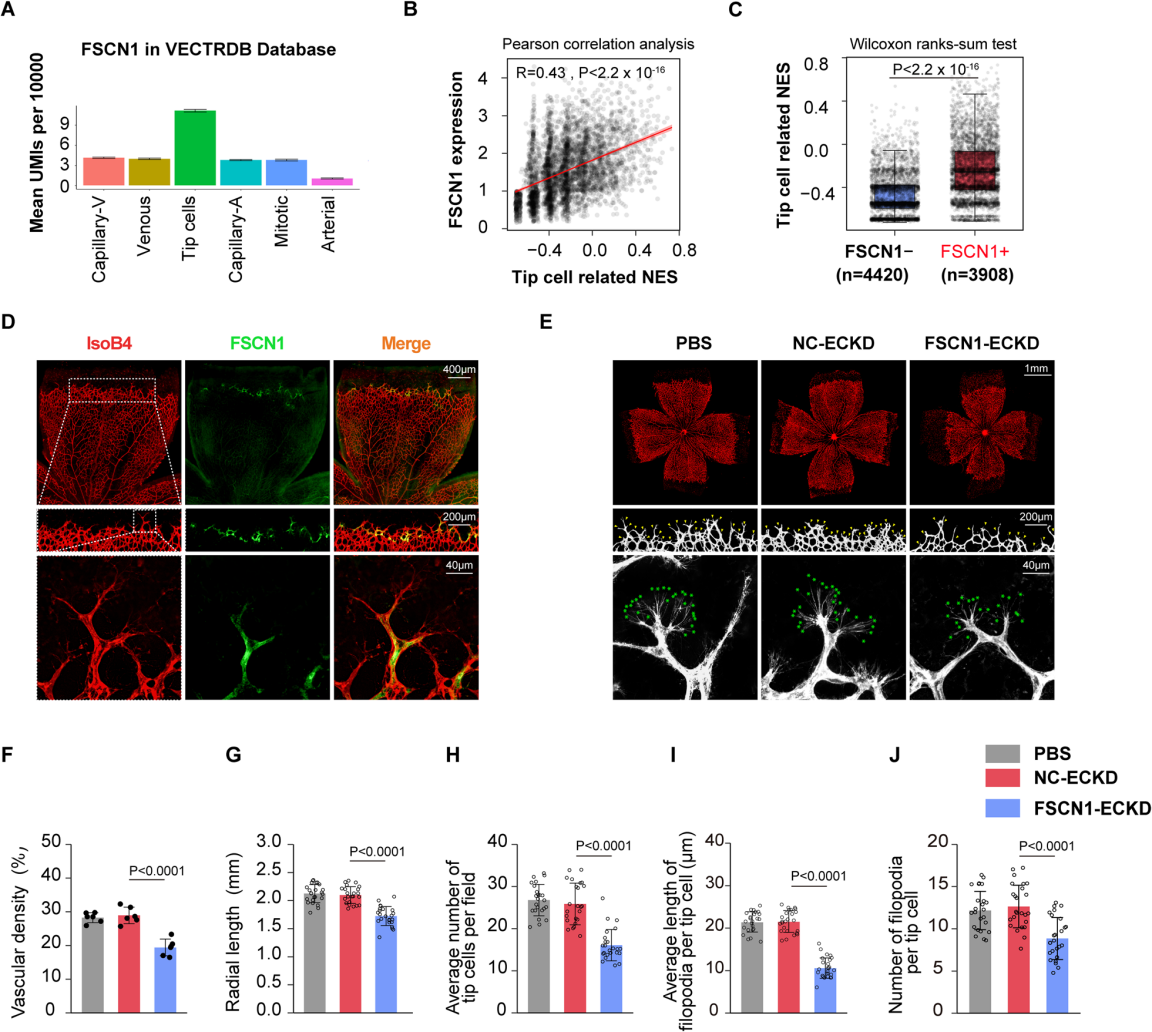
FSCN1 was located in the anterior end of the retinal vascular, and knockdown of FSCN1 could regulates tip cells behaviors in vivo.** A** The histogram of FSCN1 expression level in distinct endothelial subsets is downloaded from the Vascular Endothelial Cell Trans-omics Resource Database (VECTRDB) (https://markfsabbagh.shinyapps.io/vectrdb/).B A scatter plot correlation analysis is performed using Pearson correlation test to test the association between FSCN1 expression and tip cell-related normalized enrichment scores (NES). The scatter plot's correlation R-value and P-value is label on the top right. (n = 8,328 cells)**. C** Tip cell-related normalized enrichment scores (NES) are compared between FSCN1 + ECs and FSCN1- ECs. Results are presented as mean ± SEM, statistical analyses were performed using Wilcoxon ranks-sun test. (FSCN1 + ECs, n = 3,908 cells vs FSCN1- ECs, n = 4,420 cells). **D** Colocalization of FSCN1 and the anterior end of the retinal vascular (IsoB4: red; FSCN1: green). Scale bar: 40 μm. (n = 4 independent experiments). **E** The vitreous body of C57BL/6 J mice pups are injected with PBS, AAVsig-TIE-FSCN1 shRNA (“FSCN1-ECKD”) or AAVsig-TIE-NC shRNA (“NC-ECKD”) at P1, and collected retinas five days after injection. The group injected PBS is taken as the control group. Retinal flat is stained with IsoB4, and red areas highlighted vascular tufts. Top panels represent the vascular radial length and density of developing retinas (Scale bar: 1 mm). (n = 6 mice per group); Middle and down panels represent the number of tip cells (yellow arrow) (Scale bar:200 μm) at the vascular front of developing retinas (n = 24 fields from 6 mice per group), the number and length of filopodia (green asterisk) (Scale bar: 40 μm) respectively. (n = 12 fields from 6 mice per group). **F** Quantification of the density of developing retinal vascular. Results are presented as mean ± SEM, statistical analyses were performed using One-way ANOVA with Bonferroni's post hoc test. (n = 6 mice per group). **G** Quantification of the length of developing retinal vascular radial. Results are presented as mean ± SEM, statistical analyses were performed using One-way ANOVA with Bonferroni's post hoc test. (n = 24 directions from 6 mice per group). **H** Quantification of the average number of tip cells per field. Results are presented as mean ± SEM, statistical analyses were performed using One-way ANOVA with Bonferroni's post hoc test. (n = 24 fields from 6 mice per group). **I** Quantification of the average length of filopodia per tip cell. Results are presented as mean ± SEM, statistical analyses were performed using Kruskal–Wallis with Bonferroni's post hoc test. (n = 12 fields from 6 mice per group). **J** Quantification of the number of filopodia per tip cell. Results are presented as mean ± SEM, statistical analyses were performed using One-way ANOVA with Bonferroni's post hoc test. (n = 12 fields from 6 mice per group)

Previous studies have established that the formation of filopodia is crucial for the specialization of tip cells, a distinct process whereby activated endothelial cells continuously compete for a leading position to direct the direction of sprouting vessels during sprouting angiogenesis [17, 51, 52]. Given our data suggesting a critical role for FSCN1 in filopodia formation in vitro (Fig. 4), we hypothesized that FSCN1 may regulate tip cell behaviors. Using the Vascular Endothelial Cell Trans-omics Resource Database (VECTRDB) [53], we obtained the FSCN1 expression levels (in UMIs per 10000) in the developing brain EC single-cell expression dataset, which revealed that FSCN1 is highly expressed in endothelial tip cells (Fig. 5A). Notably, FSCN1 + ECs displayed higher expression levels of tip cell-enriched genes, such as DLL4, FLT4, and ADM, compared to FSCN1- ECs (Fig. 3D). Additionally, we calculated the GSVA-score for a tip cell-enriched gene set (tip cell related NES) in each endothelial cell and found that FSCN1 expression correlated strongly with tip cell-related NES (Fig. 5B). FSCN1 + ECs also displayed higher tip cell-related NES than FSCN1- ECs (Fig. 5C). These results suggest a robust correlation between FSCN1 and endothelial tip cells.

Given the superficial distribution of the retinal capillary network, the developing retina provides an excellent platform for visualizing tip cells. Therefore, we investigated whether FSCN1 is expressed at the angiogenic front of retinal vessels. Our hypothesis was confirmed when we observed that the immunofluorescent signal of FSCN1 was localized to the anterior end of the vascular in the P6 retina (Fig. 5D), which co-localized with DLL4, a marker for vascular tip cells (Additional file 2: Figure S2A), while no immunofluorescent signal of FSCN1 was detected in P1 and P28 retinas (Additional file 2: Figure S2B).

To determine the role of FSCN1 in tip cell behavior in vivo, we designed AAVsig-TIE-FSCN1 shRNA (“FSCN1-ECKD”) or AAVsig-TIE-NC shRNA (“NC-ECKD”) and intravitreally injected them into C57BL/6 J mice pups at P1 to achieve FSCN1 endothelial conditional knockdown in developing mouse retinas (identified by western blot in primary retinal vascular endothelial cells with ~ 45% knockdown efficiency) (Additional file 1: Figure S1B). Endothelial conditional knockdown of FSCN1 in C57BL/6 J mice from P1 resulted in impaired retinal angiogenesis at P6. Specifically, developing retinas with FSCN1-ECKD exhibited 18.83% and 31.31% reduced vascular radial length and density, respectively, compared to those with NC-ECKD (Fig. 5E-G). Furthermore, tip ECs at the vascular front of FSCN1-ECKD developing retinas exhibited reduced number, accompanied by decreased filopodia length and number, compared with those with NC-ECKD (Fig. 5E, H-J). In conclusion, our findings suggest that FSCN1 is essential for filopodia formation and controls tip cell specialization during sprouting angiogenesis.

### Conditional knockdown of FSCN1 inhibits pathological ocular neovascularization in vivo

Most studies on FSCN1 have focused on targeting carcinoma. However, it is not yet known whether FSCN1 can be a potential therapeutic target to inhibit pathological ocular neovascularization. To determine the role of FSCN1 in pathological retinal neovascularization in vivo, we injected FSCN1-ECKD or NC-ECKD into the vitreous body of oxygen-induced retinopathy (OIR) mice at P12 to achieve FSCN1 endothelial conditional knockdown in OIR mouse retinas (5 days after injection). We identified this knockdown through western blot analysis in primary retinal vascular endothelial cells (~ 52% knockdown efficiency) (Additional file 1: Figure S1 C).

**Fig. 6:**
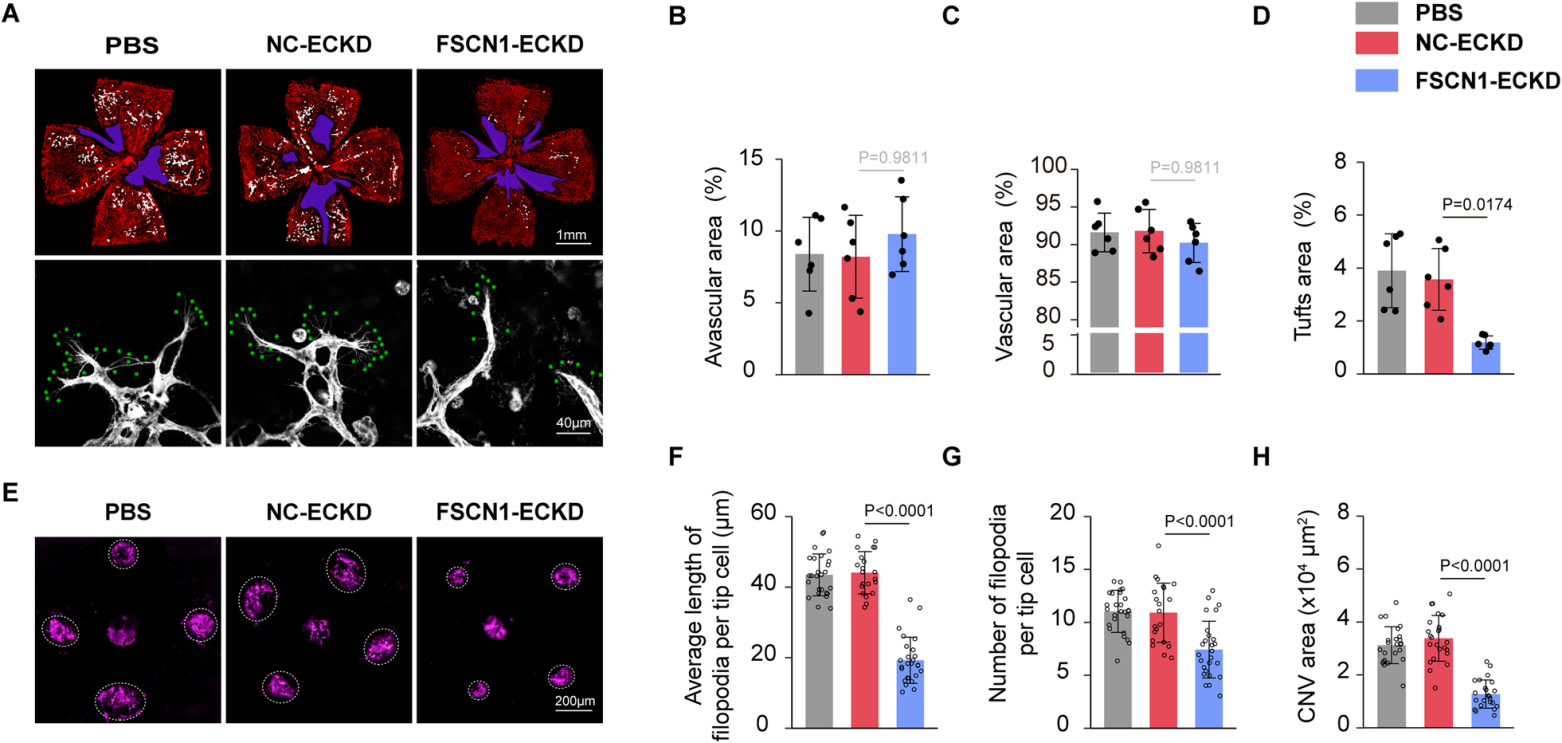
Knockdown of FSCN1 could represses pathological ocular neovascularization in vivo **A** The vitreous body of oxygen-induced retinopathy (OIR) C57BL/6 J mice were injected with PBS, AAVsig-TIE-FSCN1 shRNA (“FSCN1-ECKD”) or AAVsig-TIE-NC shRNA (“NC-ECKD”) at P12, and removed retinas five days after injection (P17). The group injected PBS is taken as the control group. Top panels: [Retinal flat are stained with IsoB4.The blue regions represent central avascular areas and the white regions highlight neovascular tuft area. (Scale bar:1 mm). (n = 6 mice per group)]; [Down panels represent the number and length of filopodia (green asterisk) (Scale bar:40 μm) at the leading edge of retinal vascularization. (n = 12 fields from 6 mice per group)]. **B** Quantification of central avascular area in P17 OIR. Results are presented as mean ± SEM, statistical analyses were performed using One-way ANOVA with Bonferroni's post hoc test. (n = 6 mice per group). **C** Quantification of peripheral vascular area in P17 OIR. Results are presented as mean ± SEM, statistical analyses were performed using One-way ANOVA with Bonferroni's post hoc test. (n = 6 mice per group). **D** Quantification of neovascular tuft area in P17 OIR. Results are presented as mean ± SEM, statistical analyses were performed using Kruskal–Wallis with Bonferroni's post hoc test. (n = 6 mice per group). **E** Choroidal flat mounts of 4-week-old mice subjected one week prior to laser-CNV injected with PBS, AAVsig-TIE-FSCN1 shRNA (“FSCN1-ECKD”) or AAVsig-TIE-NC shRNA (“NC-ECKD”) stained with IsoB4 (purple). Scale bar, 200 μm. (n = 6 mice per group). **F** Quantification of the average length of filopodia per tip cell. Results are presented as mean ± SEM, statistical analyses were performed using One-way ANOVA with Bonferroni's post hoc test. (n = 12 fields from 6 mice per group). **G** Quantification of the number of filopodia per tip cell. Results are presented as mean ± SEM, statistical analyses were performed using One-way ANOVA with Bonferroni's post hoc test. (n = 12 fields from 6 mice per group). **H** Quantification of CNV surface area. Results are presented as mean ± SEM, statistical analyses were performed using One-way ANOVA with Bonferroni's post hoc test. (n = 24 laser dots from 6 mice per group)

Conditional knockdown of FSCN1 in retinal ECs significantly reduced retinal neovascularization with a decrease in neovascular tuft area, but the avascular regions of the central and peripheral part of the retina were not affected (Fig. 6A-D). Furthermore, the filopodia length and number were significantly decreased at the leading edge of vascularization with FSCN1-ECKD (Fig. 6F, G), as described in developing retinas (Fig. 5E).

We also used a laser-induced choroidal neovascularization (CNV) model to determine the role of FSCN1 in pathological choroidal neovascularization in vivo. FSCN1-ECKD or NC-ECKD was intravitreally injected into adult C57BL/6 J mice (6-week-old) following laser photocoagulation, and its effect was verified by western blot analysis in primary choroidal vascular endothelial cells (~ 65% knockdown efficiency) (Additional file 1: Figure S1 D). Conditional knockdown of FSCN1 in choroidal ECs significantly repressed the CNV area by over 50% compared to the negative control at 7 days after CNV induction (Fig. 6E, H).

These findings suggest that FSCN1 knockdown represses pathological ocular neovascularization in vivo, indicating FSCN1 as an attractive target for developing new regimens of anti-angiogenic drug therapy.

### NP-G2-044, a FSCN1 inhibitor, in vitro cytotoxicity study

NP-G2-044 was initially identified as a FSCN1 inhibitor and has demonstrated anti-tumor potency in a phase Ia clinical trial (Clinicaltrials.gov NCT03199586). Currently, it is being evaluated in a phase I/II clinical trial (Clinicaltrials.gov NCT05023486). Based on the anti-angiogenic potency of FSCN1 knockdown observed in our data, we anticipate that NP-G2-044 may function not only as an anti-metastatic drug for malignant tumors but also as a promising drug for pathological ocular neovascularization.

**Fig. 7:**
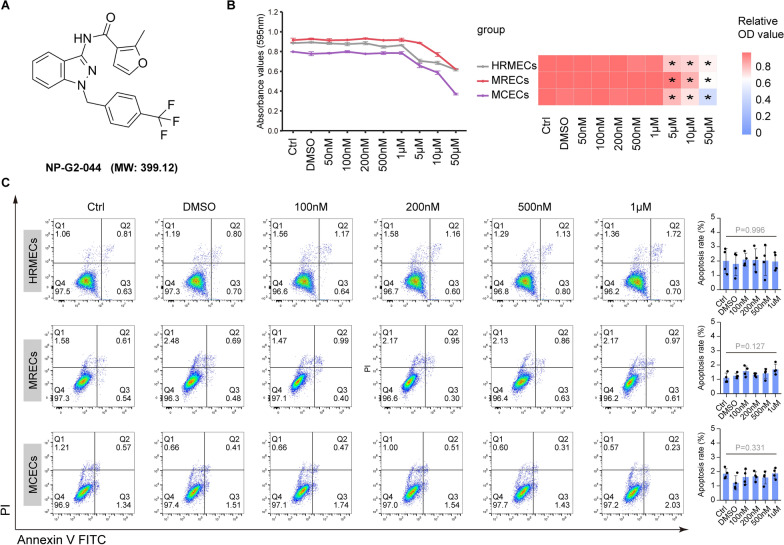
FSCN1 inhibitor (NP-G2-044) with the range of 0.05-1 μmol /L was nontoxic to critical ocular cells. After 48 h of treatment with different concentrations of NP-G2-044, the cells were examined for apoptosis.** A** The chemical structure and molecular formula of the NP-G2-044 is provided from PubChem database. **B** Cytotoxicity of different concentrations (50 nM-50 μM) of NP-G2-044 measured by MTT assay on different cell lines. MTT activity of different cell lines is read at OD 595 nm and indicated by the line chart (left panel). The OD values are corrected by the control group and indicated by the heatmap (right panel), (asterisk indicate that the concentration is statistically significant compared with Ctrl group), (n = 3 per group, data pooled from 3 independent experiments). **C** Flow cytometry assay for cell apoptosis in endothelial cell lines (HRMEC, MREC, MCEC). Both early-apoptotic (AV + and PI –) and late-apoptotic (AV + and PI –) cells were included in the analyses. Quantification of the rate of apoptosis. Results are presented as mean ± SEM, statistical analyses were performed using One-way ANOVA. (n = 4 per group, data pooled from 4 independent experiments)

The chemical structure and molecular formula of NP-G2-044 (Compound CID: 91844684) are available in the PubChem database (Fig. 7A). To assess the in vitro cytotoxicity of NP-G2-044, we treated different endothelial cell lines (HRMECs, MRECs, MCECs) with various drug concentrations for 48 h and performed the MTT cytotoxicity assay to test its effect on cell viability. Our results indicated that NP-G2-044 had no significant cytotoxicity on HRMEC, MREC, and MCEC cells at concentrations ranging from 0.05 to 1 μM. However, it was toxic to endothelial cell lines at concentrations above 1 μM (Fig. 7B, C). We further investigated the effect of NP-G2-044 on cell apoptosis using Annexin V-FITC/PI staining and flow cytometry (FCM) analysis. Our results showed no significant difference in the rate of apoptosis induction in endothelial cell lines at concentrations of 0.05 to 1 μM (Fig. 7C). Collectively, we determined that non-toxic concentrations of NP-G2-044 in vitro range from 0.05 to 1 µmol/L.

### In vivo toxicity and pharmacokinetic studies of NP-G2-044 following oral administration

**Fig. 8:**
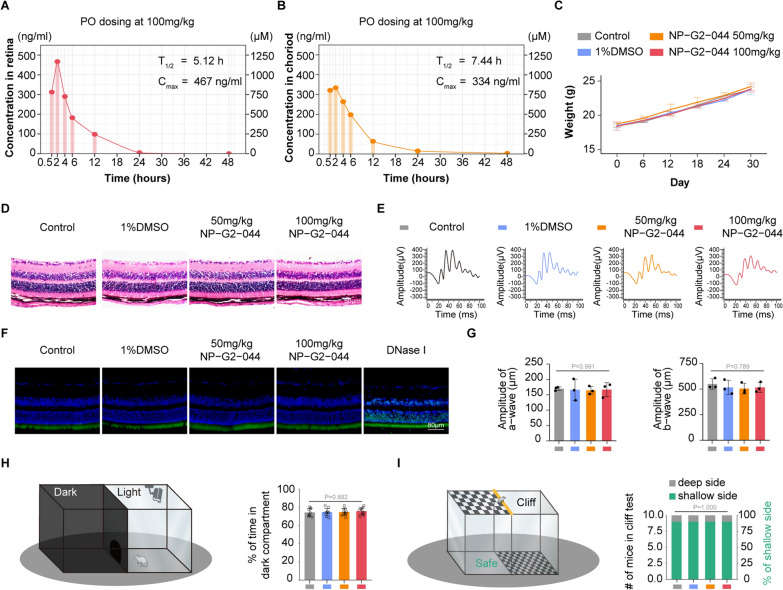
The toxicity, visual function and pharmacokinetics studies of NP-G2-044 in vivo after oral administration. **A**, **B** The ocular concentration of NP-G2-044 in the retinal and choroidal tissues at the different time points (~ 0 h, 0.5 h, 2 h, 4 h, 6 h, 12 h, 24 h and 48 h) analyzed by HPLC–MS/MS to identify the preclinical pharmacokinetic study following a single-dose oral administration (100 mg/kg) to mice. T_1/2:_ half-life. **C** Quantification of the weight of mice at different time points after long-term oral administration (2 times daily for 30 days in the dose of 50 mg/kg or 100 mg/kg). (n = 4 per group, data pooled from 3 independent experiments). **D** H&E staining shows the morphology of retinal and choroidal to mice after different treatments with PBS,1%DMSO, 50 mg/kg NP-G2-044 or 100 mg/kg NP-G2-044 oral administration twice daily for 30 days. Oral administration of PBS is considered as the control group. (Scale bar: 80 μm). (n = 4 per group, data pooled from 4 independent experiments). **E** Dark-adapted Electroretinography (ERG) assessed retinal function after long-term oral administration. [a-wave (derived from photoreceptors) reflects outer retinal function, while the b-wave (derived from müller and bipolar cells) reflects inner retinal function.]. (n = 3 per group, data pooled from 3 independent experiments). **F** TUNEL staining assays evaluate the apoptosis of cells in the retina and choroid after long-term oral administration. (Scale bar: 80 μm). (n = 4 per group, data pooled from 4 independent experiments). **G** Quantification of the amplitude of a − wave (µm) and b − wave (µm) in ERG. Results are presented as mean ± SEM, statistical analyses were performed using One-way ANOVA. (n = 3 per group, data pooled from 3 independent experiments). **H** The light/dark transition test assesses visual sensitivity in the mice after long-term oral administration. Schematic diagram of the visual cliff tests (left panel). Quantification of the percentage of time in dark compartment (right panel). Results are presented as mean ± SEM, statistical analyses were performed using One-way ANOVA. (n = 4 independent experiments). (n = 10 per group, data pooled from 3 independent experiments). **I** The visual cliff test assesses visual depth perception in the mice after long-term oral administration. Schematic diagram of the visual cliff tests (left panel). Quantification of the percentage of choosing the shallow side (right panel). Results are presented as mean ± SEM, statistical analyses were performed using Fisher’s exact test. (n = 4 independent experiments). (n = 10 per group, data pooled from 3 independent experiments)

Previous studies have demonstrated the high oral bioavailability of NP-G2-044 in plasma [44]. However, for ophthalmic drugs, their therapeutic effectiveness largely depends on their permeability across the blood-retina barrier. In this study, we applied an HPLC–MS/MS assay to investigate the drug's penetration from the circulation, across the blood-retina barrier, into the posterior eye segment. A preclinical pharmacokinetic study was performed using the established HPLC–MS/MS method, following a single-dose oral administration (100 mg/kg) to mice. The ocular concentration of NP-G2-044 was measured in the retinal and choroidal tissues at different time points (0 h, 0.5 h, 2 h, 4 h, 6 h, 12 h, 24 h, and 48 h), showing sufficient retina concentration (Cmax467 ng/ml|1170 µM, T_1/2_5.12 h) and choroid concentration (Cmax334 ng/ml|836 µM, T_1/2_7.44 h) (Fig. 8A, B). Retinal and choroidal concentrations were approximately one-third to one-half of the plasma concentration from previous trials [44]. Based on these initial investigations, it appears that NP-G2-044 is rapidly absorbed after oral administration, penetrates the blood-retina barrier well, and has a half-life of 5.12–7.44 h, allowing for two to three times daily dosing of 100 mg/kg in mice.

To evaluate the toxicity of NP-G2-044, particularly its toxicity to ocular tissues, a series of in vivo toxicity evaluations were conducted, including long-term oral administration (twice daily for 30 days at doses of 50 mg/kg or 100 mg/kg) to assess chronic toxicity. IsoB4 staining assay was conducted to evaluate the impact of NP-G2-044 on mature retinal vasculature, and the results showed no alteration in the density of retinal blood vessels (Additional file 3: Figure S3A). Dark-adapted Electroretinography (ERG) was used to assess retinal function: the a-wave (derived from photoreceptors) reflects outer retinal function, while the b-wave (derived from müller and bipolar cells) reflects inner retinal function. ERG recordings pointed to normal retinal function in treated mice (Fig. 8E–G). The visual acuity was determined by behaviorally testing, including Visual Cliff Test and Light/dark Transition Test. No visual impairment was observed in treated mice (Fig. 8H, I). Tissue apoptosis was evaluated with TUNEL staining assays, and fundus structure was examined with H&E staining. Retinal tissues showed no apoptosis and no morphological alterations in treated mice. (Fig. 8D,F). Furthermore, systemic toxic effects were evaluated by measuring body weight (Fig. 8C) and examining injury to vital organs, including the heart, liver, spleen, lung, and kidney, with histopathologic analysis. No significant systemic toxicity was observed in these experiments (Additional file 3: Figure S3B). Based on these results, we conclude that NP-G2-044 is non-toxic both in major organs of the body and in ocular tissues.

### NP-G2-044 shows individual efficacy to attenuate pathological ocular neovascularization and increases the efficacy of anti-VEGF therapy

**Fig. 9:**
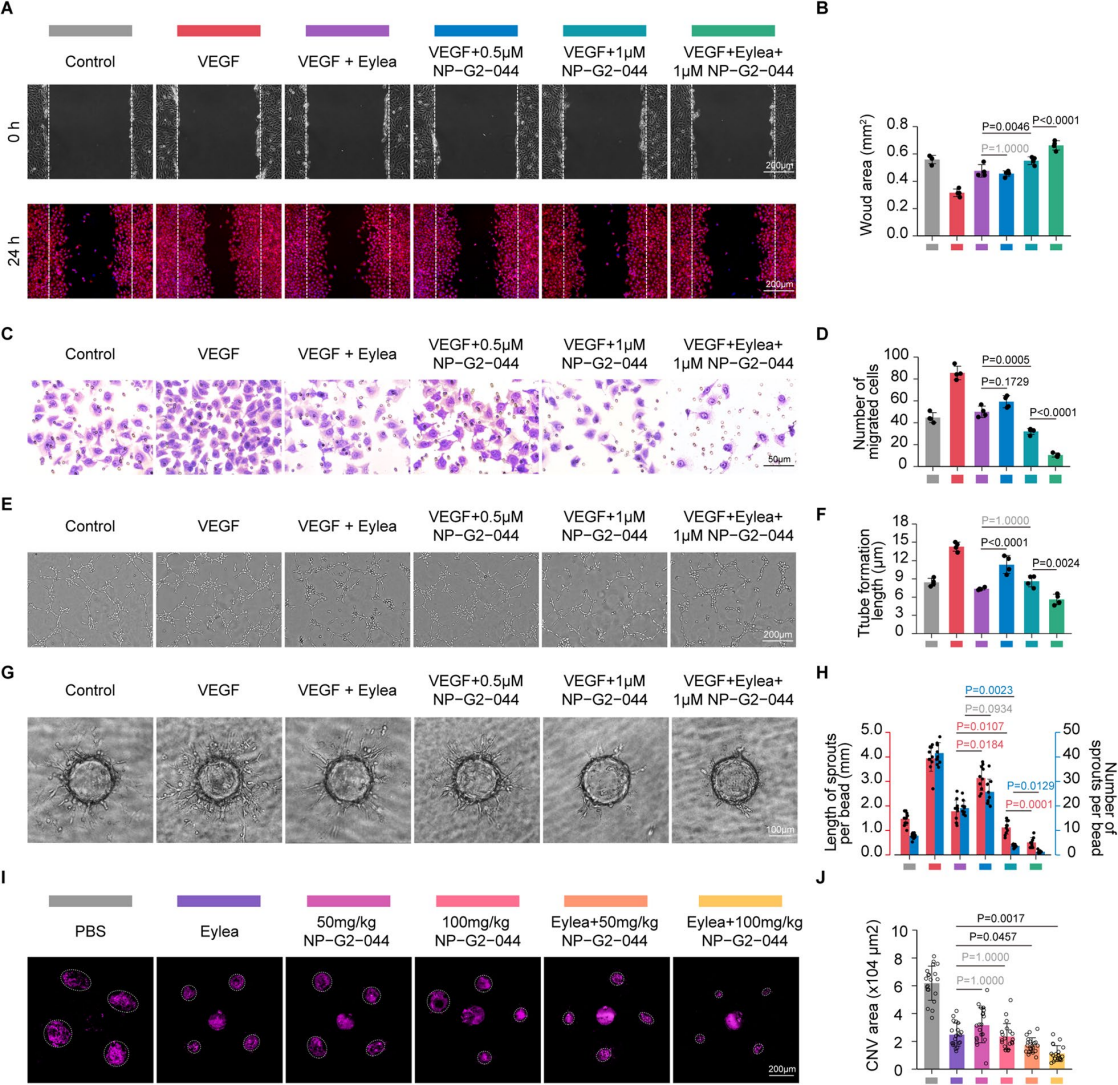
NP-G2-044 could attenuate pathological ocular neovascularization and increases the efficacy of anti-VEGF therapy in vivo and in vitro. HRMECs are added in VEGF, VEGF + Eylea, VEGF + 0.5 μM NP − G2 − 044, VEGF + 1 μM NP − G2 − 044, VEGF + Eylea + 1 μM NP − G2 − 044 or left untreated. The group without any treatment is taken as the control group. **A** Wound scratching assays of HRMECs under different treatments. Scale bar: 200 µm. [ rhodamine-conjugated phalloidin: red (to visualize the actin cytoskeleton); DAPI: blue (to visualize nuclei)]. (n = 4 per group, data pooled from 4 independent experiments). **B** Quantification of wound area. The top panels represent the initial state of the wound area at 0 h, and the down panels represent the area after a period of 24 h of hypoxic migration. Results are presented as mean ± SEM, statistical analyses were performed using One-way ANOVA with Bonferroni's post hoc test. (n = 4 per group, data pooled from 4 independent experiments). **C** Transwell assays of HRMECs under different treatments. Scale bar: 50 µm. (n = 4 per group, data pooled from 4 independent experiments). **D** Quantification of migrated cells. Results are presented as mean ± SEM, statistical analyses were performed using One-way ANOVA with Bonferroni's post hoc test. (n = 4 per group, data pooled from 4 independent experiments). **E** Tube formation Assay of HRMECs under different treatments. Scale bar: 200 µm. (n = 4 per group, data pooled from 4 independent experiments). **F** Quantification of tube formation length. Results are presented as mean ± SEM, statistical analyses were performed using One-way ANOVA with Bonferroni's post hoc test. (n = 4 per group, data pooled from 4 independent experiments). **G** Three-dimensional (3D) Bead Sprouting Assay reveals the in vitro sprouting capabilities of HRMECs under different treatments. Scale bar: 100 µm. (n = 10 per group, data pooled from 4 independent experiments). **H** Quantification of length and number of sprouts per bead. Results are presented as mean ± SEM, statistical analyses were performed using Kruskal–Wallis with Bonferroni's post hoc test. (n = 10 per group, data pooled from 4 independent experiments). **I** Choroidal flat mounts of 4-week-old mice subjected one week prior to laser-CNV injected with PBS or Eylea whether added different concentrations of NP − G2 − 044 or not stained with IsoB4 (purple). The vitreous body of all mice (except group injected with Eylea) are injected with PBS and the group injected with only PBS is taken as the control group. Scale bar, 200 μm. (n = 20 laser dots from 5 mice per group). **J** Quantification of CNV surface area. Results are presented as mean ± SEM, statistical analyses were performed using One-way ANOVA with Bonferroni's post hoc test. (n = 20 laser dots from 5 mice per group). (n = 20 laser dots from 5 mice per group)

It was imperative to ascertain the anti-angiogenic efficacy of NP-G2-044 in vitro and in vivo using non-toxic concentrations. The major stimulus for pathological ocular neovascularization is vascular endothelial growth factor (VEGF), a key angiogenic actor secreted by RPE cells and glial cells in response to hypoxia, which mediates angiogenic functions by binding to its cognate receptors on endothelial cells. Therefore, exogenous VEGF was added to the culture medium of HRMECs to mimic the angiogenic conditions of in vitro cultures. In vitro experiments, including tube formation assay, wound scratching assay, transwell assay, and three-dimensional (3D) bead sprouting assay, were performed and indicated that NP-G2-044 resulted in a significant dose-dependent reduction of VEGF-induced angiogenic effects (Fig. 9A–H). To compare the anti-angiogenic efficacy of NP-G2-044 with conventional anti-VEGF drugs, Eylea (a therapeutic anti-VEGF drug) was used. Furthermore, we found similar efficacy between Eylea (1 mg/mL) and a high dose of NP-G2-044 (1 µmol/L), as well as better efficacy in combination with Eylea and NP-G2-044 (Fig. 9A-H). Of note, the anti-angiogenic efficacy of combination therapy completely repressed and even far exceeded the angiogenic effect induced by exogenous VEGF, suggesting that NP-G2-044 might act in an alternative pathway, non-redundant to VEGF angiogenic pathways.

Next, we investigated the anti-angiogenic effect and therapeutic efficacy of NP-G2-044 in vivo using a laser-induced CNV model. Oral administration of NP-G2-044 (twice daily in the dose of 50 mg/kg or 100 mg/kg) was used to test in vivo anti-angiogenic efficacy alone or in combination with intravitreal Eylea injection. The in vivo CNV model results for NP-G2-044 confirmed its oral activity in attenuating pathological ocular neovascularization, in accordance with the in vitro study (Fig. 9I, J). In particular, compared to intravitreal Eylea injection, combined therapy (oral administration of NP-G2-044 plus vitreous injection of Eylea) revealed a 2.5-fold decrease in the CNV area at day 7 post-laser (Fig. 9I, J). Overall, both in vitro and in vivo experiments demonstrated significant angiogenesis inhibition induced either by NP-G2-044 or Eylea alone and more impressive efficacy induced by combined treatment.

### NP-G2-044 and anti-VEGF combination overcome anti-VEGF resistance

**Fig. 10:**
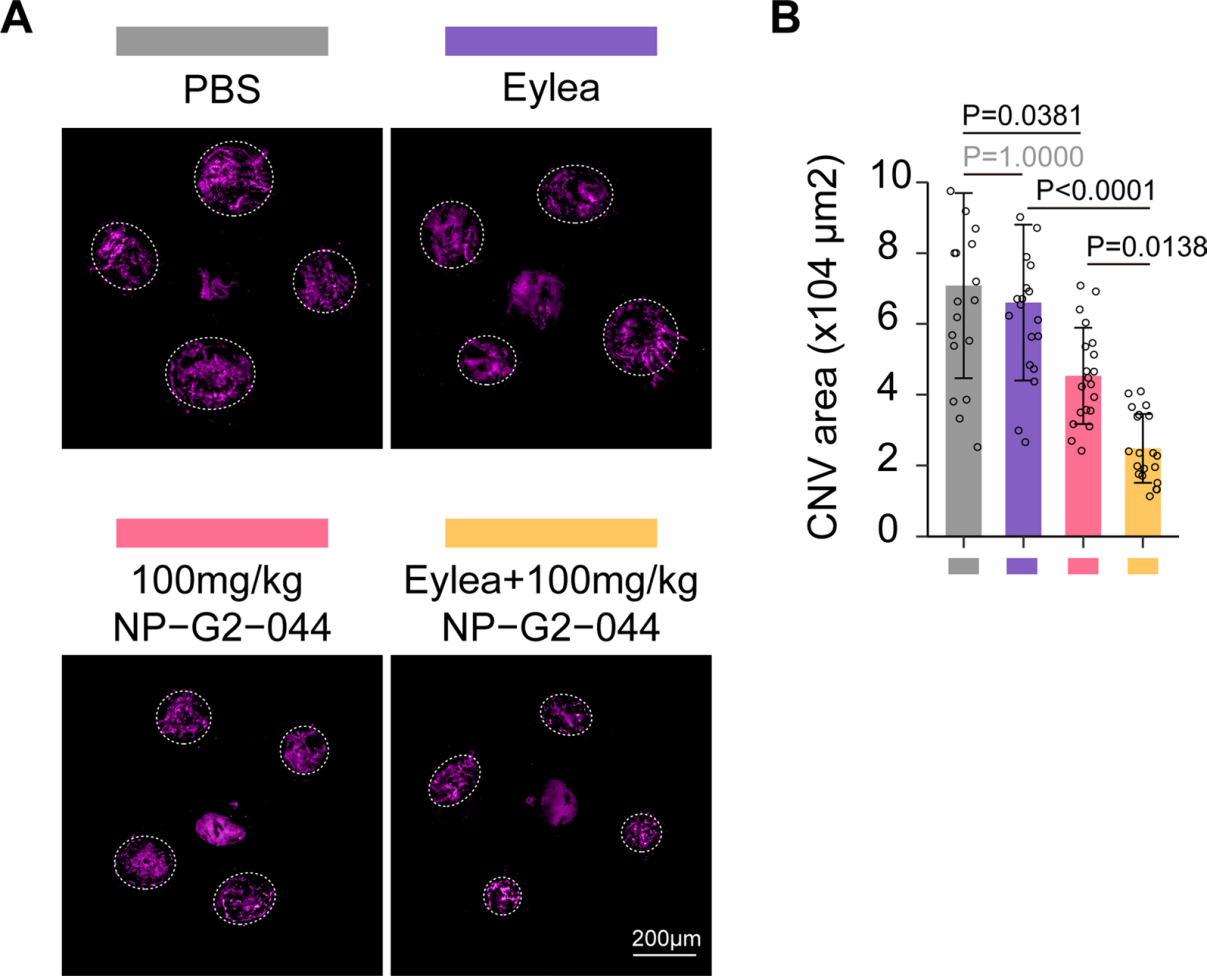
NP-G2-044 combined with anti-VEGF drugs could improve anti-VEGF resistance.** A** Choroidal flatmounts of old CNV mice (around 12-months-old) treated with Eylea, 100 mg/kg NP − G2 − 044 or Eylea + 100 mg/kg NP − G2 − 044 were stained with IsoB4 (purple). The vitreous body of all mice (except group injected with Eylea) are injected with PBS and the group injected with only PBS is taken as the control group. Scale bar, 200 μm. (n = 20 laser dots from 5 mice per group). **B** Quantification of CNV surface area. Results are presented as mean ± SEM, statistical analyses were performed using One-way ANOVA with Bonferroni's post hoc test. (n = 20 laser dots from 5 mice per group)

It has been proposed by Zhu et al. that laser-induced CNV in old mice tends to have a higher incidence of anti-VEGF resistance [54]. Therefore, we established the CNV model in approximately 12-month-old mice as described above. We observed no significant decrease in CNV area after vitreous injection of Eylea in old CNV mice (Fig. 10A, B), confirming the successful establishment of a feasible and reproducible anti-VEGF resistant model. Excitingly, oral administration of NP-G2-044 (100 mg/kg given twice daily) showed potent angiogenesis inhibition, whereas vitreously applied Eylea was ineffective (Fig. 10A, B). Furthermore, combined therapy exhibited over a two-fold decrease compared to NP-G2-044 alone (Fig. 10A, B), suggesting a synergistic effect of NP-G2-044 in increasing the sensitivity of anti-VEGF therapy. This synergistic effect could be attributed to NP-G2-044’s ability to anti-angiogenesis in a VEGF-independent manner (see below for more details). Therefore, NP-G2-044 could be combined with current anti-VEGF therapy to prevent or rescue resistance.

### NP-G2-044 inhibits FSCN1/actin binding, leading to reduced nuclear localization of YAP and depressed CDC42 GTP activity

**Fig. 11:**
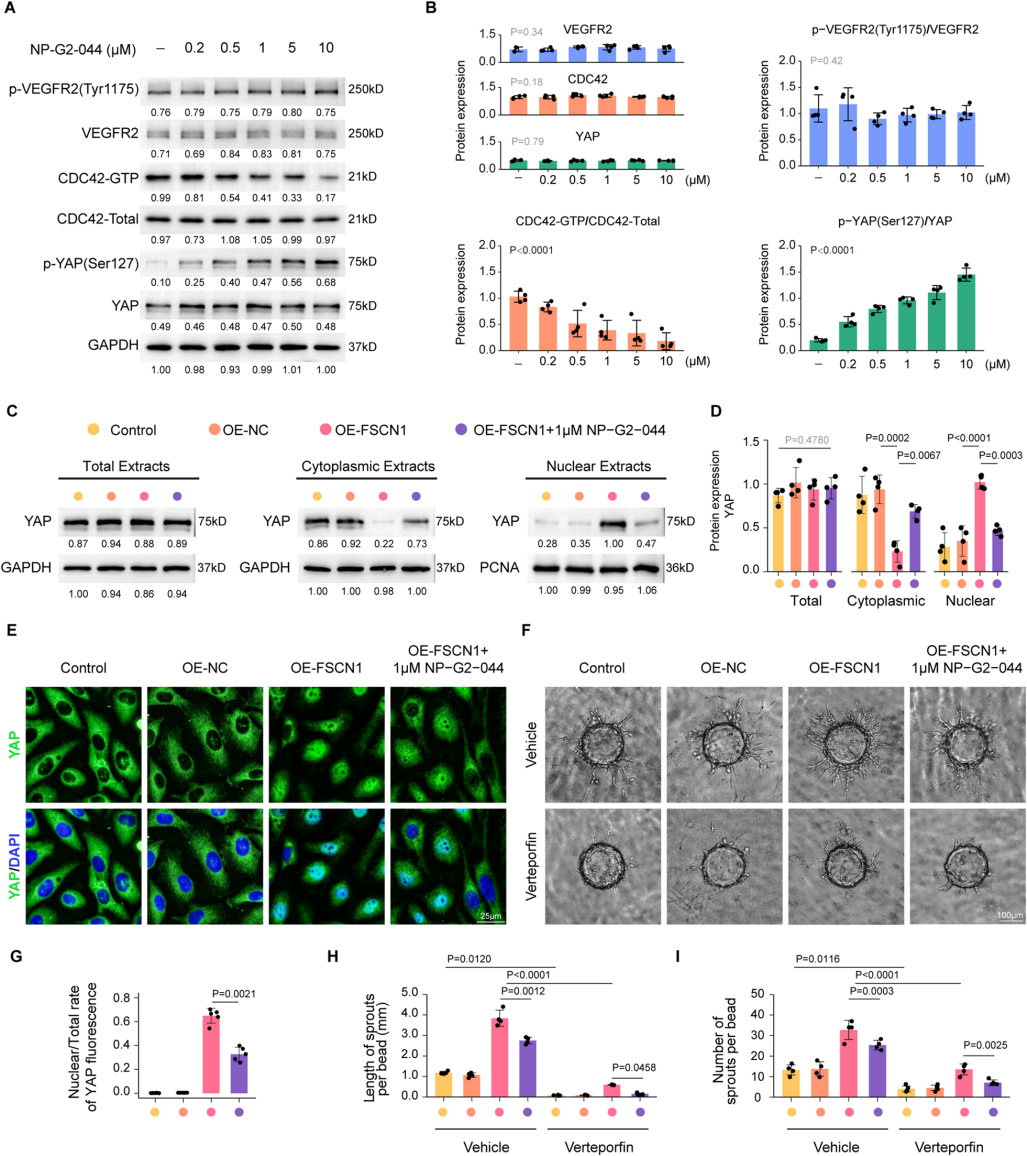
NP-G2-044 inhibited FSCN1 binding to F-actin, resulting in reduced nuclear entry of YAP and inhibited CDC42 GTP activity.** A** Western blotting assays test the protein expression of p-VEGFR2(Tyr1175), VEGFR2, CDC42-GTP, CDC42-Total, p − YAP(Ser127), YAP and GAPDH in HRMECs after treatment with VEGF-A (20 ng/ml) for 5 min and different concentrations of NP − G2 − 044. (n = 4 independent experiments). **B** Densitometric quantitation of Western blot band intensity shown in A. Results are presented as mean ± SEM, statistical analyses were performed using One-way ANOVA. (n = 4 independent experiments). **C** HRMECs were transfected with OE-NC, OE-FSCN1, OE-FSCN1 added with 1 μM NP − G2 − 044 or left untreated for 24 h. The group without any treatment is taken as the control group (D, F). Total, cytoplasmic, nuclear extracts from the resulting cells are analyzed by WB for YAP expression. (n = 4 independent experiments). **D** Densitometric quantitation of Western blot band intensity shown in **C**. Results are presented as mean ± SEM, statistical analyses were performed using One-way ANOVA with Bonferroni's post hoc test. (n = 4 independent experiments). **E** Localization of YAP is demonstrated by immunofluorescence. Scale bar, 50 μm. (n = 5 per group, data pooled from 5 independent experiments). **F** HRMECs were transfected with OE-NC, OE-FSCN1, OE-FSCN1 added with 1 μM NP − G2 − 044 or left untreated in the presence or absence of 5 μM verteporfin for 24 h, DMSO as a vehicle control. Three-dimensional (3D) Bead Sprouting Assay reveals the in vitro sprouting capabilities of HRMECs under different treatments. Scale bar: 100 µm. (n = 4 per group, data pooled from 4 independent experiments). **G** Quantification of the rate of Nuclear/Total YAP fluorescence. Results are presented as mean ± SEM, statistical analyses were performed using Kruskal–Wallis with Bonferroni's post hoc test. (n = 5 per group, data pooled from 5 independent experiments). **H**, **I** Quantification of length and number of sprouts per bead. Results are presented as mean ± SEM, statistical analyses were performed using One-way ANOVA with Bonferroni's post hoc test. (n = 4 per group, data pooled from 4 independent experiments)

Recently, the co-crystal structure of the NP-G2-029 and FSCN1 complex was elucidated, providing a structural basis for the selective functional inhibition of FSCN1 [40]. NP-G2-044 can modify the conformation of FSCN1 to block its actin-binding sites, thereby inhibiting its actin-binding activity [40]. Through immunoprecipitation in HRMECs, the interaction between FSCN1 and actin was clearly demonstrated, and the interaction was noticeably impeded by varying concentrations of NP-G2-029 in a dose-dependent manner (Additional file 4: Figure S4A). We initially examined whether NP-G2-044 regulates angiogenesis via the VEGF signaling pathway. VEGF can rapidly activate VEGFR2, with a peak of VEGF activation occurring at 2–5 min. After stimulating HRMECs with VEGF (20 ng/mL) for 5 min, the expression of total VEGFR2 and phosphorylated VEGFR2 (p-VEGFR2) were evaluated by immunoblotting, and no significant changes were detected across different concentrations of NP-G2-029, indicating that NP-G2-044 operates independently of the VEGF/VEGFR2 cascade (Fig. 11A, B).

Given the critical role of CDC42 (a Rho-family GTPase) in tip cell filopodia formation [52], we further examined the expression of total CDC42 and CDC42-GTP (the active form of the GTPase), showing that NP-G2-044 markedly reduced the gradient of CDC42 GTP activity in HRMECs (Fig. 11A, B). Besides, Prior studies have established that YAP dephosphorylation induces nuclear localization of Yap, which drives tip cell behaviors via activating molecules involved in the reorganization of the actin cytoskeleton [55, 56]. Based on the pivotal function of FSCN1 in actin cytoskeleton rearrangement and filopodia formation, we also investigated whether FSCN1 and its inhibitor NP-G2-044 affect phosphorylation and subcellular localization of YAP. As expected, with increasing inhibitor concentration, we observed a progressive, step-wise elevation in the level of YAP phosphorylation (Fig. 11A, B). After transfecting an FSCN1 overexpression plasmid into HRMECs (Additional file 1: Figure S1E), we observed that FSCN1 overexpression inhibited YAP phosphorylation, which was restored after NP-G2-044 administration (Additional file 4: Figure S4B). Immunofluorescence microscopy also revealed that FSCN1 overexpression caused a shift from cytoplasmic to nuclear distribution of YAP, whereas NP-G2-044 resulted in prominent nuclear exclusion of YAP and partially reversed the effect induced by FSCN1 overexpression (Fig. 11E, G). The western blot results were consistent with the immunofluorescence results, showing abnormal YAP nuclear accumulation in FSCN1-overexpressed HRMECs and potent inhibition of YAP nucleocytoplasmic shuttling with NP-G2-044 (Fig. 11C, D). After the administration of Verteporfin (an inhibitor of YAP), HRMECs exhibited defects in their sprouting process, while also effectively preventing excessive sprouting resulting from the overexpression of FSCN1. Notably, the application of a combined treatment consisting of NP-G2-044 and Verteporfin resulted in a more significant inhibition of HRMECs sprouting (Fig. 11F, H and I). Overall, NP-G2-044 operates in an alternative pathway that is non-redundant to the VEGF pathway, and may represent a synergistic treatment strategy to eliminate resistance to anti-VEGF therapy in pathological ocular neovascularization.

## Disscussion

Ocular neovascularization is a significant pathological characteristic of various prevalent ocular diseases, such as retinopathy of prematurity (ROP), proliferative diabetic retinopathy (PDR), and age-related macular degeneration (AMD) [1]. Vascular endothelial growth factor (VEGF) blockade is currently the first-line therapy for ocular neovascularization; however, it is partially limited by adverse effects and resistance [5, 6]. Therefore, there is a great demand for anti-angiogenic therapeutics with new targets to overcome these limitations. Pathological neovascularization is initiated by the formation of new vessel sprouts from the existing vasculature, which is mainly mediated by tip cells [8]. Hence, a better understanding of tip cell behaviors could guide future anti-angiogenic research. The development of tip cell-targeting agents is a promising direction. In this study, we investigated the key role of FSCN1 in tip cells during sprouting angiogenesis and hypothesized that pharmacologically targeting FSCN1 would be a potential strategy for the development of novel anti-angiogenic therapeutics in pathological ocular neovascularization.

In this study, we defined the new roles of FSCN1 in the behavior of tip cells during sprouting angiogenesis in ocular tissues, including physiological and pathological angiogenesis. FSCN1 is a highly conserved actin-bundling protein that is essential for filopodia formation in tip cells by cross-linking actin filament bundles [35, 57]. Therefore, it is reasonable to hypothesize that FSCN1 plays a crucial role in tip cell behavior. Our research, based on single-cell RNA (scRNA) analysis and validated experiments, revealed a striking finding that FSCN1 is specifically expressed in vascular endothelial cells, particularly tip cells, in the retina and choroid. Previous studies have reported that FSCN1, which is localized in filopodia underneath the plasma membrane [35], is associated with increased cell motility, cell invasion, and aggressive tumor behavior [31]. Similarly, FSCN1 knockdown impaired the migratory and sprouting capability of vascular endothelial cells in vitro hypoxia models and resulted in reduced filopodia outgrowth. Furthermore, conditional knockdown of FSCN1 in endothelial cells disrupted angiogenesis in the mouse retina and choroid by impeding endothelial specialization into tip cells and limiting filopodia outgrowth.

Recent advances in anti-angiogenic therapies targeting tip cells have shown promising results. Activated Ang1/Tie2 and Dll4/NOTCH signaling induce and restrict the tip cell phenotype, respectively [14, 58]. Previously, Rennel et al. developed a neutralizing monoclonal antibody (mAb) to specifically target Ang2, an antagonistic ligand against Ang1/Tie2 signaling. It was shown that intravitreally injected Ang2 mAb has a potentially therapeutic effect in models of retinal and choroidal neovascularization [59]. Alternatively, Yan et al. developed a soluble human Dll4 (hD4R) to repress ocular neovascularization by effectively triggering Notch signaling in endothelial cells [60]. Additionally, our previous study showed that targeting circular RNA-MET by inhibiting endothelial tip cell specialization can yield promising effects for anti-angiogenesis treatment [17]. However, there are currently no pharmacological agents specifically targeting tip cells that have been developed for clinical use in the treatment of neovascular ocular diseases. Given the high expression of FSCN1 in angiogenic tip cells, we hypothesized that NP-G2-044, an orally active FSCN1 inhibitor, could be developed as a novel tip cell-targeting agent against pathological ocular neovascularization. Our results demonstrated that oral NP-G2-044 alone potently attenuates laser-induced choroidal neovascularization, suggesting the potential utility of NP-G2-044 in the treatment of pathological ocular neovascularization.

How do FSCN1 and NP-G2-044 regulate the angiogenic phenotype of tip cells? In addition to its previously known role as an actin-bundling protein required for filopodia formation [35]. This study has highlighted the newly identified roles of FSCN1 in tip cell specialization. Recent evidence suggests that YAP is required for the proper execution of tip cell behaviors, including cytoskeletal remodeling, filopodia formation, and tip cell specialization [55, 56]. Moreover, Cdc42, a critical effector for filopodia initiation and extension, is associated with YAP nuclear translocation [55]. As demonstrated in this study, overexpression of FSCN1 triggered the shift of YAP's distribution from cytoplasmic into nuclear, where it is activated to provoke a downstream transcriptional program promoting tip cell behaviors. Based on both immunofluorescence and western blot analysis, we conclude that FSCN1 is a new regulator of YAP nucleocytoplasmic shuttling to control tip cell behaviors. Furthermore, our data also revealed that NP-G2-044 suppressed CDC42 GTP activity and reduced nuclear accumulation of YAP, finally leading to attenuated ocular neovascularization.

The characterization of expression patterns can be of crucial importance for discovering novel drug targets. We found that FSCN1 was specifically expressed at the vascular front of retina at postnatal day 6 and silenced in the adult mouse retina. Moreover, FSCN1 was highly expressed in various pathological ocular neovascularization models. This observation is in agreement with previous findings demonstrating the expression of FSCN1 in healthy adult mice is negligible to null [61], making FSCN1 a promising and safe target for the development of therapeutic agents. In fact, a variety of toxicology studies have shown that the safety of NP-G2-044 in various species, including mice, rats, dogs and humans; no toxicity was observed even in ultra-high dose of 1000 mg/kg/day in animal models [42]. However, little is known about the toxicity of NP-G2-044 to ocular tissues. Here, we performed the first comprehensive toxicity assays in terms of ophthalmology, with detailed insights on cytotoxicity, apoptosis, histomorphology, visual acuity and retinal function, and concluded that NP-G2-044 was also no-toxic to adult mouse ocular tissues.

The absorption and penetration of orally administered drugs are critical factors that determine their bioavailability. The eye has unique physiological barriers, such as the outer and inner blood-retinal barriers, which create challenges for drug delivery to the posterior segment of the eye, including the choroid and retina [62]. It is well-known that the blood-retinal barrier typically permits the permeation of small molecules with high lipid solubility [63]. NP-G2-044 is a small, lipid-soluble molecule that has the potential to pass through this natural barrier. Previous pharmacodynamic studies have shown that NP-G2-044 is rapidly absorbed orally into plasma and eliminated with an average T_max_ of 1–6 h and T_1/2_ of 3–6 h in mice [44]. Additionally, in humans (Clinicaltrials.gov NCT03199586), the drug was well-absorbed orally into plasma with T_max_ of 4 h and T_1/2_ of 20–24 h. Our data further revealed that the drug was also well-penetrated into ocular tissues (retinas and choroids) with a T_max_ of approximately 2 h and T_1/2_ of 5.12–7.44 h in mice. Wang et al. deduced that NP-G2-044 could be extensively penetrated in peripheral tissue, based on the estimated steady-state volume of distribution [44]. Our evidence supports this deduction, as sufficient concentrations of NP-G2-044 were detected in the retina and choroid (retina C_max_ ~ 467 ng/mL|1170 µM, choroid C_max_ ~ 334 ng/mL|836 µM), which were approximately equivalent to half of the plasma concentration [44]. Notably, due to species diversity, a significantly longer T_1/2_ was observed in humans (about threefold longer than in mice), suggesting that the efficacy of NP-G2-044 may be underestimated in mouse models. We also found that ocular tissues had a slightly longer T_1/2_ of NP-G2-044 than plasma, indicating a slower clearance in ocular tissues and longer retention time. Based on the above-mentioned evidence, we conclude that NP-G2-044 is a safe and well-permeable, orally administered anti-angiogenic drug for pathological ocular neovascularization.

According to previous real-world studies, 20–40% of patients with wet age-related macular degeneration (wAMD) and 15–20% of those with diabetic retinopathy (DR) fail to respond, or only partially respond to anti-VEGF therapy [64]. This wide resistance to anti-VEGF therapy has prompted the search for novel therapeutic targets. Previous reports have suggested the role of FSCN1 in the acquisition of chemoresistance in human cancers, such as paclitaxel, doxorubicin, and gefitinib resistance [65–67]. Thus, it was postulated that drugs targeting FSCN1 may address the challenge of anti-VEGF resistance. Although there is no direct evidence of the correlation between FSCN1 and anti-VEGF resistance, this is partially illustrated by the fact that a significant reduction in CNV lesion was found upon oral application of NP-G2-044 in an anti-VEGF-resistant CNV mouse model, whereas no significant effects were observed upon application of Eylea alone. Several proposed mechanisms for anti-VEGF resistance in neovascular eye disease include: (1) enhanced protection by pericytes in vessel stabilization to alleviate exogenous VEGF dependence [68], (2) exacerbated inflammation and infiltration of microglia/macrophages [69], (3) increased neovascular fibrosis, which acts as a resorption barrier [70], (4) remnant empty sleeves of vascular basement membrane [71], and (5) endothelial cell adaptation to activate alternative angiogenic signaling besides the VEGF axis [72]. Oral NP-G2-044 is as effective as Eylea in suppressing laser-induced CNV, and this response is independent of VEGF actions on VEGFR2. On the other hand, NP-G2-044 directly disrupts the formation of filopodia, avoiding the compensation of other pro-angiogenic factors, and is therefore more specific than anti-VEGF drugs. This may explain why NP-G2-044 performed better than Eylea in the anti-VEGF-resistant CNV mouse model. Targeting FSCN1 with NP-G2-044 represents an alternative, combinative therapeutic strategy for pathological ocular neovascularization, exerting synergistic effects to increase efficacy, as well as additional effects against anti-VEGF resistance.

Overall, our study provides promising insights into the potential benefits of NP-G2-044 for patients with neovascular eye disease. There are several reasons for this. Firstly, oral administration of drugs is the preferred method of treatment due to its low invasiveness, minimal infection risk, good patient compliance, and ease of administration [73]. However, there are currently no FDA-approved oral drugs for the treatment of pathological ocular neovascularization. Therefore, the potential of NP-G2-044 as an oral treatment option for this condition is particularly noteworthy. Secondly, NP-G2-044 offers an alternative treatment option for patients with pathological ocular neovascularization who do not respond to anti-VEGF drugs. Our animal models have demonstrated that NP-G2-044 can increase anti-VEGF efficacy and overcome anti-VEGF resistance. If these results are confirmed in further clinical studies, it would be excellent news for these patients. However, it is important to acknowledge that our study has some limitations and that further research is required. For example, our study only focused on the effect of FSCN1 on ocular angiogenesis, and we need to investigate its role in other neural cells, including neuronal and glial cells, in regulating ocular phenotype. Furthermore, although we have revealed the regulatory role of FSCN1 in CDC42 GTP activity and YAP nucleocytoplasmic shuttling, the exact mechanism by which FSCN1 affects tip cell specification is not yet clear. In vivo and in vitro investigations will be necessary to elucidate this. We also need to verify whether anti-VEGF resistant patients express higher levels of FSCN1, despite scRNA-seq data indicating that its expression is upregulated in wAMD patients. Finally, the most important aspect is that targeting FSCN1 with NP-G2-044 has demonstrated a significant suppression of pathological neovascularization in laser-induced CNV models, and this finding warrants further investigation in clinical settings.

## Materials and methods

Additional information can be found in the Additional file 5.

### Processing and analysis of published scRNA-seq data

Publicly available single-cell RNA sequencing (scRNA-seq) data for oxygen-induced retinopathy (OIR) and age-related macular degeneration (AMD) were retrieved from the NCBI public Gene Expression Omnibus (GEO) database with accession codes GSE150703 [49] and GSE135922 [50], respectively. Raw counts were downloaded and processed using the R package Seurat (version 3.0) to create a normalized Seurat object with quality control standards. These standards included min.cells = 3, min.features = 100, mito.ratio < 0.20, nFeature_RNA >  = 300, nFeature_RNA < 5000, respectively. Highly variable genes (top 5000) were identified using the FindVariableFeatures function, which performed principal component analysis (PCA) to select the top 30 principal components for further cluster analysis. Unsupervised hierarchical clustering was performed using the FindClusters function with an appropriate resolution and visualized using Uniform Manifold Approximation and Projection (UMAP) projections. Cluster annotation for endothelial cells was performed by evaluating the expression density of known endothelial cell (EC) marker genes (CDH5 and PECAM1) in UMAP projections, using the R package Nebulosa (version 1.3.0).

For further analysis, EC clusters were selected for the ratio and level analysis of Fascin Actin-Bundling Protein 1 (FSCN1) expression between various groups. Based on the scRNA-seq count data, the EC clusters were divided into two groups: an FSCN1-positive (“FSCN1 + ”) EC cluster and an FSCN1-negative (“FSCN1-”) EC cluster. To further understand the main biological effect of FSCN1 in the EC cluster, differentially expressed genes (DEGs) between the two clusters were identified using the FindMarkers function. Gene set enrichment analysis (GSEA) was performed with the R package clusterProfiler (version 3.18.1) to annotate the DEGs with known biological processes (BP). Gene set variation analysis (GSVA) was used with the R package GSVA (version 1.32.0) to calculate the normalized enrichment score (NES) of a tip cell-enriched gene set collected from the literature (Table 1).

**Table 1:** A tip cell-enriched gene set consisting of 18 genes

ID	Gene symbol	Description
1	ESM1 [16]	Endothelial cell specific molecule 1
2	KDR [13]	Kinase insert domain receptor
3	PDGFB [51]	Platelet derived growth factor subunit B
4	FLT4 [74]	Fms related receptor tyrosine kinase 4
5	NRP1 [52]	Neuropilin 1
6	CXCR4 [15]	C-X-C motif chemokine receptor 4
7	RGCC [50]	Regulator of cell cycle
8	EDNRB [75]	Endothelin receptor type B
9	DLL4 [14]	Delta like canonical Notch ligand 4
10	ADM [18]	Adrenomedullin
11	APLN [76]	Apelin
12	ANGPT2 [10]	Angiopoietin 2
13	LAMB1 [76]	Laminin subunit beta 1
14	PLAUR [53]	Plasminogen activator, urokinase receptor
15	MCAM [77]	Melanoma cell adhesion molecul
16	MMRN2 [78]	Multimerin 2
17	KCNE3 [79]	Potassium voltage-gated channel subfamily E regulatory subunit
18	LCP2 [53]	Lymphocyte cytosolic protein

### Plasmids, shRNA lentivirus and adeno-associated virus establishment

For overexpression plasmids, the human full-length FSCN1 gene was cloned into a pEX-4 (pGCMV/MCS/T2A/EGFP/Neo) vector (GenePharma) to construct FSCN1 overexpression plasmids (“oeFSCN1”). Empty pEX-4 plasmid was used as negative control (NC) for the overexpression experiments (“oeNC”). HRMEC cells, at approximately 70% confluence, were serum starved for 4 h prior to incubation with Lipofectamine 3000 (Invitrogen, L3000-015) and overexpression plasmids for 5 h. 24 h following transfection, cells were harvested and subjected to the next analysis. In in vitro knockdown experiment, cells were divided into the following groups: the control group (added with DMEM), oeNC group (transfected with oeNC), oeFSCN1 group (transfected with oeFSCN1).

Lentiviral vectors containing short hairpin RNA (shRNA) targeting the FSCN1 sequence (LV-shFSCN1 (“shFSCN1”)) or scrambled sequence (LV-shNC (“shNC”)) were constructed with titers of 1 × 10^9^ virus particles/ml, and the target sequence was identical to following sequence in adeno-associated viruses (AAV). To stably knockdown the expression level of FSCN1 in HRMEC cells, cells were incubated with shRNA lentiviral particles for 4 h in the presence of 8 µg/mL polybrene (Sigma-Aldrich, TR-1003) before changing the media. To select stably resistant cells, cells were grown continuously in media supplemented with 1 µg/ml puromycin for 14 days. In in vitro knockdown experiment, cells were divided into the following groups: the control group (added with DMEM), shNC group (added with shNC), shFSCN1 group (added with shFSCN1).

Adeno-associated viruses (AAV) under the control of the endothelial-specific promoter (TIE) and an AAVsig serotype (SIGYPLP-modified AAV2) were uesd to induce efficiently endothelial-specific knockdown in mice retinas and choroids. The AAV expressing a short hairpin RNA (shRNA) targeting the FSCN1 sequence (AAVsig-TIE-FSCN1 shRNA (“FSCN1-ECKD”)) or scrambled sequence (AAVsig-TIE-NC shRNA (“NC-ECKD”)) was constructed with titers of 1 × 10^12^ virus particles/ml, and the target sequence was as follows: FSCN1: 5'-GCGCCUACAACAUCAAAGATT-3', NC: 5'-GCGCGATAGCGCTAATAATTT-3'. In in vivo experiment, animals were divided into the following groups: the control group (intravitreally injected with 1 µl PBS), NC-ECKD group (intravitreally injected with 1 µl NC-ECKD), FSCN1-ECKD group (intravitreally injected with 1 µl FSCN1-ECKD).

### Preparation of NP-G2-044

NP-G2-044 (MCE, HY-125506) was purchased from MedChemExpress. The stock solution was prepared by dissolving NP-G2-044 in DMSO (Fisher Chemical, BP231-1) at a maximum solubility concentration of 200 mM and stored at − 80 °C. Prior to use in vitro experiments, the stock solution was diluted with cell culture DMEM media to the indicated concentrations (50 nM, 100 nM, 200 nM, 500 nM, 1 µM, 5 µM, 10 µM, 50 µM), and added to cell culture dishes. Prior to use in in vivo oral experiments, the stock solution of NP-G2-044 in DMSO was diluted in corn oil to achieve a gavage dose of 50 mg/kg or 100 mg/kg body weight.

### HPLC–MS/MS analysis

A high performance liquid chromatography-tandem mass spectrometry (HPLC–MS/MS) method is developed for determination of compound concentrations in the plasma or tissue according to the method described below. The fresh tissues (retinas or choroids) were collected from 6-week-old C57BL/6 J mice with a single-dose oral administration of 100 mg/kg NP-G2-044 at the different time points (~ 0 h, 0.5 h, 2 h, 4 h, 6 h, 12 h, 24 h and 48 h) and stored at -80 ℃. When the sample collection period was completed, samples was dissolved in a 100 µl mixture of acetonitrile and water (1:1, v/v), continuously vortexed for 15 min, sonicated (40Hkz) for 5 min in a ultrasonic cleaner, and then centrifuged at 13,200 rpm for 4 min; 50 µl supernatants were aspirated into a clean 200 µl microfuge tube and the proteins were removed by adding 150 µl protein precipitant containing an internal standard followed by centrifugation at 13,200 rpm for 4 min; the remaining supernatants were collected and analyzed in a HPLC–MS/MS system (Shimadzu LC-20AD and API 3200MD TRAP). The liquid phase conditions used were: column: MSLab C18 (50 × 4.6 mm 2.5 µm); column temperature: 50 ℃; flow rate: 1 mL/min; injection volume: 5 µL; mobile phase A: aqueous phase: Water (ammonium acetate); mobile phase B: organic phase: acetonitrile (ammonium acetate); and gradient elution: 0.01–0.90 min, (5% A: 95% B); 0.91–3.20 min, (70% A: 30% B); 3.21–4.00 min, (95% A: 5% B); 4.01–6.00 min, (5% A: 95% B). The following pharmacokinetics parameters were determined in mice retina and choroid: peak ocular tissue concentration (C_max_), and the half-time of ocular tissue concentration (T_1/2_).

### Quantitative real-time PCR (qRT-PCR)

Total cellular mRNA was extracted using TRIzol reagent (Invitrogen, A33250) by chloroform layering and isopropanol precipitation, and then reverse transcribed into cDNA using HiScript III RT SuperMix for qPCR (+ gDNA wiper) Kit (Vazyme, A211). Quantitative real-time PCR (qRT-PCR) was performed with PowerUP**™** SYBR**™** Green Master Mix (Applied Biosystems,A25742) on a PikoReal 96 Real-Time PCR System (Thermo Fisher Scientific,TCR0096) with the following specifically designed PCR primer sequences: mouse FSCN1 Forward Primer 5ʹ-CACAGGCAAATACTGGACGGT-3ʹ and Reverse Primer 5ʹ-CCACCTTGTTATAGTCGCAGAAC-3ʹ; mouse ACTB Forward Primer 5ʹ-ATTCCTATGTGGGCGACGAG-3ʹ and Reverse Primer 5ʹ-TCTCCATGTCGTCCCAGTTG-3ʹ. The qRT-PCR conditions were: 7 min at 95 ℃, followed by 40 cycles of 95 ℃ for 5 s and 60 ℃ for 30 s.

### Western blot

Total protein content was extracted from cells or tissues using RIPA lysis buffer (Beyotime, P0013B), supplemented with a mixture of protease inhibitors (Roche, 11697498001). The protein concentration was determined using Pierce™ BCA Protein Assay Kit (Thermo Fisher Scientific, 23225), following the manufacturer’s protocols. The protein concentration was adjusted to 1 mg/ml using 5 × SDS-PAGE sample loading buffer (Beyotime, p0015), based on the BCA standard curve. Proteins were then separated using sodium dodecyl sulfate polyacrylamide gel electrophoresis (SDS-PAGE) in 1 × running buffer (14.4 g glycine, 2.9 g Tris base, and 1 g SDS per 1 L dd water). The proteins were transferred to a 0.45 μm polyvinylidene fluoride (PVDF) membrane (Millipore, IPVH0010) in 1 × transfer buffer (7.2 g glycine, 1.45 g Tris base, and 20% methanol per 1 L dd water) and then blocked using 5% skimmed milk. The membranes were then incubated with the corresponding primary and secondary antibodies, and the target protein bands were visualized using an Enhanced Chemiluminescence kit (Thermo Fisher Scientific, 32132).

### Antibodies

The following primary antibodies were used: rabbit anti-FSCN1 (Abcam, ab126772; IF 1:200, WB 1:1000, IP 1:100), mouse anti-GAPDH (Abcam, ab8245; WB 1:1000), mouse anti-F-actin (Abcam, ab205; WB 1:1000), rabbit anti-YAP (Cell Signaling Technology, 14074; IF 1:200, WB 1:1000), rabbit anti-p-YAP (Ser127) (Cell Signaling Technology, 4911; WB 1:1000), mouse anti-CDC42 (BD Biosciences, 610929; WB 1:500), rabbit anti-VEGFR2 (Cell Signaling Technology, 2479; WB 1:1000), rabbit anti-p-VEGFR2 (Tyr1175) (Cell Signaling Technology, 2478; WB 1:1000), goat anti-DLL4 (R&D Systems, AF1389; IF 1:200), and mouse anti-β‐actin (Millipore, A5441; WB 1:1000). The following secondary antibodies were used: goat anti-mouse HRP-conjugated antibodies (Beyotime, A0216; 1:1000) and goat anti-rabbit HRP-conjugated antibodies (Beyotime, A0208; 1:1000) were applied for WB, Alexa Fluor™ Plus 488 Anti-mouse IgG (H + L) (Abcam, ab150077; IF 1:400), and DyLight 405-AffiniPure Donkey Anti-goat IgG (H + L) (Jackson ImmunoResearch,705–475-147,1:400) was applied for immunofluorescence.

### Statistics

R statistical package were used for statistical analysis. All statistical data for each experiment were presented as means ± SD and statistical significance (P-value), detailly described in the figure legends. The two-tailed student's t-test or the Mann–Whitney U test was used to compare two groups. One-way ANOVA or Kruskal–Wallis with Bonferroni's post hoc test was used to compare multiple groups, comparing each group with every other group. Fisher’s exact test was used to compare groups in visual cliff test. A P-value of < 0.05 was considered for statistical significance.

### Supplementary Information

**Additional file 1: Figure S1. **The efficiency of plasmids, shRNA lentivirus and adeno-associated virus was evaluated by Western blot assay. **A-E** Western blot analyses and quantification of FSCN1 protein expression in HRVECs after stable knockdown of FSCN1 by shRNA lentiviral transfection (A), in primary retinal vascular endothelial cells extracted from P6 retinas injected with the AAVsig-TIE shRNA (B), in primary retinal vascular endothelial cells extracted from P17 OIR retinas injected with the AAVsig-TIE shRNA (C), in primary choroidal vascular endothelial cells extracted from choroids injected with the AAVsig-TIE shRNA (D), in HRVECs after overexpression of FSCN1 by plasmid transfection (E).Results are presented as mean ± SEM, statistical analyses were performed using One-way ANOVA with Bonferroni's post hoc test.

**Additional file 2: Figure S2. **Immunofluorescent signal of FSCN1 in retina. **A** Colocalization of FSCN1 and DLL4 in the anterior end of the retinal vascular. (IsoB4: red; FSCN1: green; DLL4: blue). Scale bar:25µm. (n = 4 independent experiments). **B** The localization of FSCN1 in retinal flatmounts (P1 and P28) was confirmed by immunofluorescence. (FSCN1: green; IsoB4: red). Scale bar:1mm. (n=4 independent experiments).

**Additional file 3: Figure S3. **Vascular examination of mature retina and histological examination of heart, liver, spleen, lung, kidney. **A** IsoB4 staining of retinal flatmounts shows the vessel density in mice subjected to various treatments including PBS, 1%DMSO, 50 mg/kg NP-G2-044, or 100 mg/kg NP-G2-044 oral administration twice daily for 30 days. Scale bar:1mm. (n=4 independent experiments). Results are presented as mean ± SEM, statistical analyses were performed using One-way ANOVA with Bonferroni's post hoc test. (n = 4 mice per group). **B** H&E staining shows the morphology of heart, liver, spleen, lung and kidney in mice subjected to various treatments including PBS, 1%DMSO, 50 mg/kg NP-G2-044, or 100 mg/kg NP-G2-044 oral administration twice daily for 30 days. Oral administration of PBS is considered as the control group. Scale bar:100 μm. (n = 4 per group, data pooled from 4 independent experiments).

**Additional file 4: Figure S4. **NP−G2−044 weakens the binding of FSCN1 to f-actin and inactivates YAP. **A** Coimmunoprecipitation (CO-IP) assays indicate the association of endogenous FSCN1 and F-actin in HRMEC cells after treatment with VEGF and different concentrations of NP−G2−044. (n=4 independent experiments). **B** Western blot assesses the protein expression of p−YAP(Ser127), YAP, FSCN1 and GAPDH in HRMECs transfected with OE-NC, OE-FSCN1, OE-FSCN1 added with 1μM NP−G2−044. Densitometric quantitation of Western blot band intensity shown in B. Results are presented as mean ± SEM, statistical analyses were performed using One-way ANOVA with Bonferroni's post hoc test. (n=4 independent experiments).

**Additional file 5 :** Additional materials and methods.

## Data Availability

The data presented in this study are available on request from the corresponding author.
