# Supplementary figures and images for: Targeting FSCN1 with an oral small-molecule inhibitor for treating ocular neovascularization
Source: J Transl Med. 2023 Aug 18;21:555. doi: 10.1186/s12967-023-04225-0 (PMC10436462; doi:10.1186/s12967-023-04225-0)

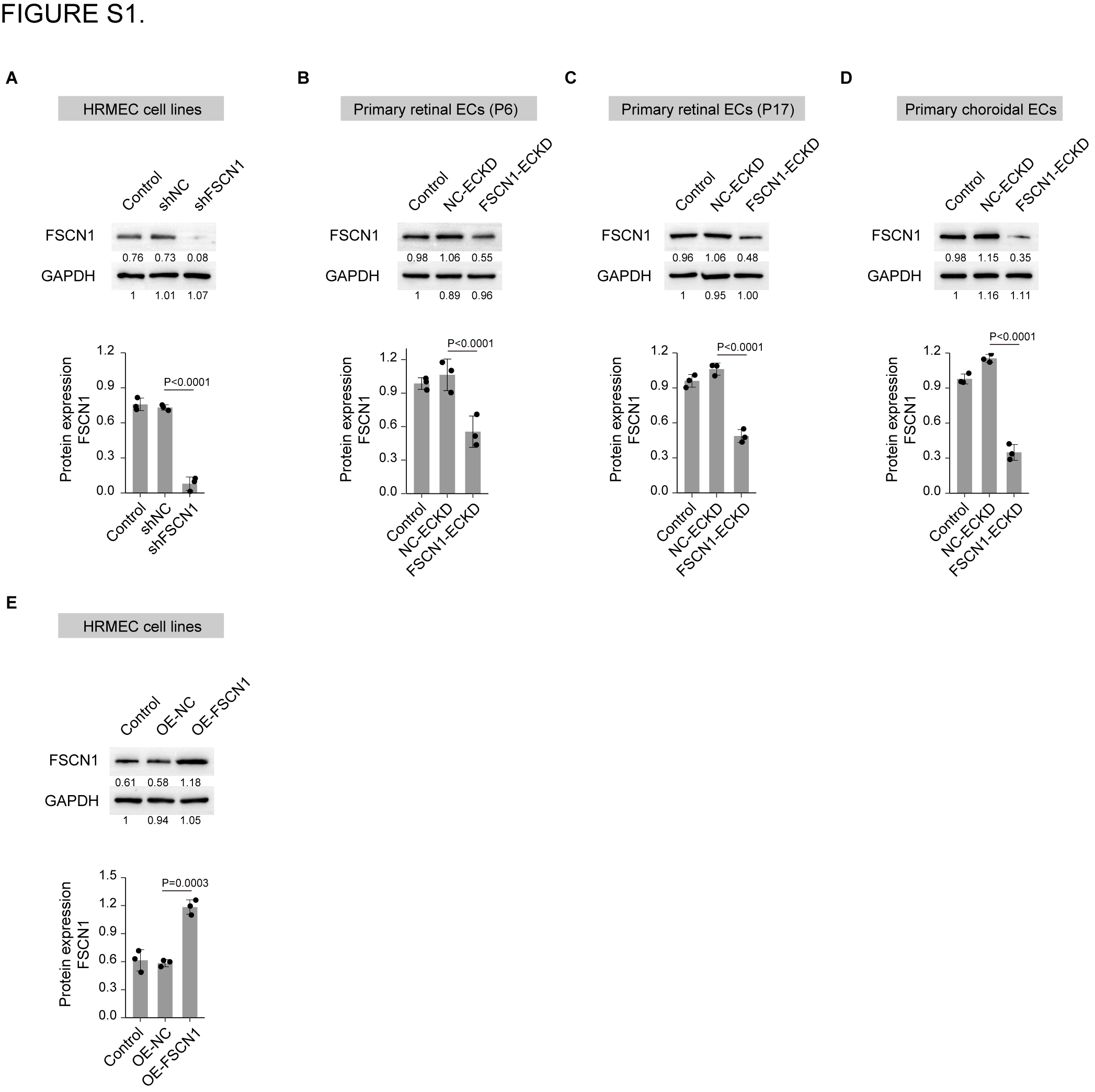

Supplement: Supplementary file 1 — Additional file 1: Figure S1. The efficiency of plasmids, shRNA lentivirus and adeno-associated virus was evaluated by Western blot assay. A-E Western blot analyses and quantification of FSCN1 protein expression in HRVECs after stable knockdown of FSCN1 by shRNA lentiviral transfection (A), in primary retinal vascular endothelial cells extracted from P6 retinas injected with the AAVsig-TIE shRNA (B), in primary retinal vascular endothelial cells extracted from P17 OIR retinas injected with the AAVsig-TIE shRNA (C), in primary choroidal vascular endothelial cells extracted from choroids injected with the AAVsig-TIE shRNA (D), in HRVECs after overexpression of FSCN1 by plasmid transfection (E).Results are presented as mean ± SEM, statistical analyses were performed using One-way ANOVA with Bonferroni's post hoc test. [file 12967_2023_4225_MOESM1_ESM.tif]

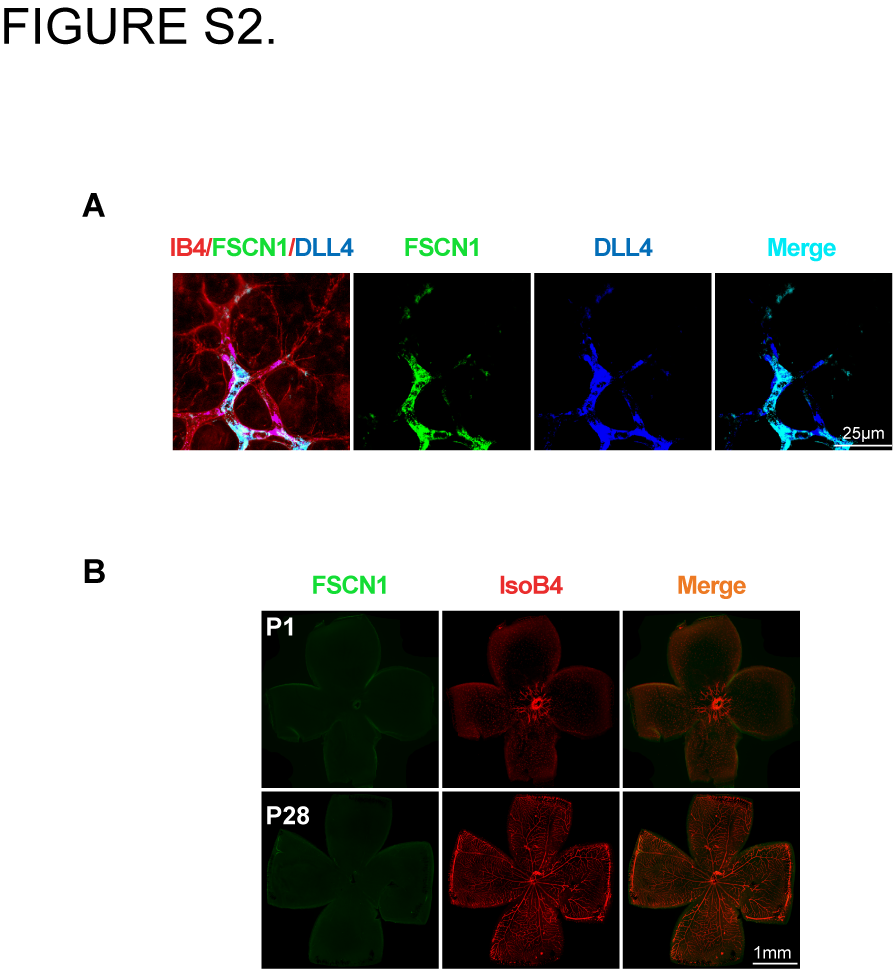

Supplement: Supplementary file 2 — Additional file 2: Figure S2. Immunofluorescent signal of FSCN1 in retina. A Colocalization of FSCN1 and DLL4 in the anterior end of the retinal vascular. (IsoB4: red; FSCN1: green; DLL4: blue). Scale bar:25µm. (n = 4 independent experiments). B The localization of FSCN1 in retinal flatmounts (P1 and P28) was confirmed by immunofluorescence. (FSCN1: green; IsoB4: red). Scale bar:1mm. (n=4 independent experiments). [file 12967_2023_4225_MOESM2_ESM.tif]

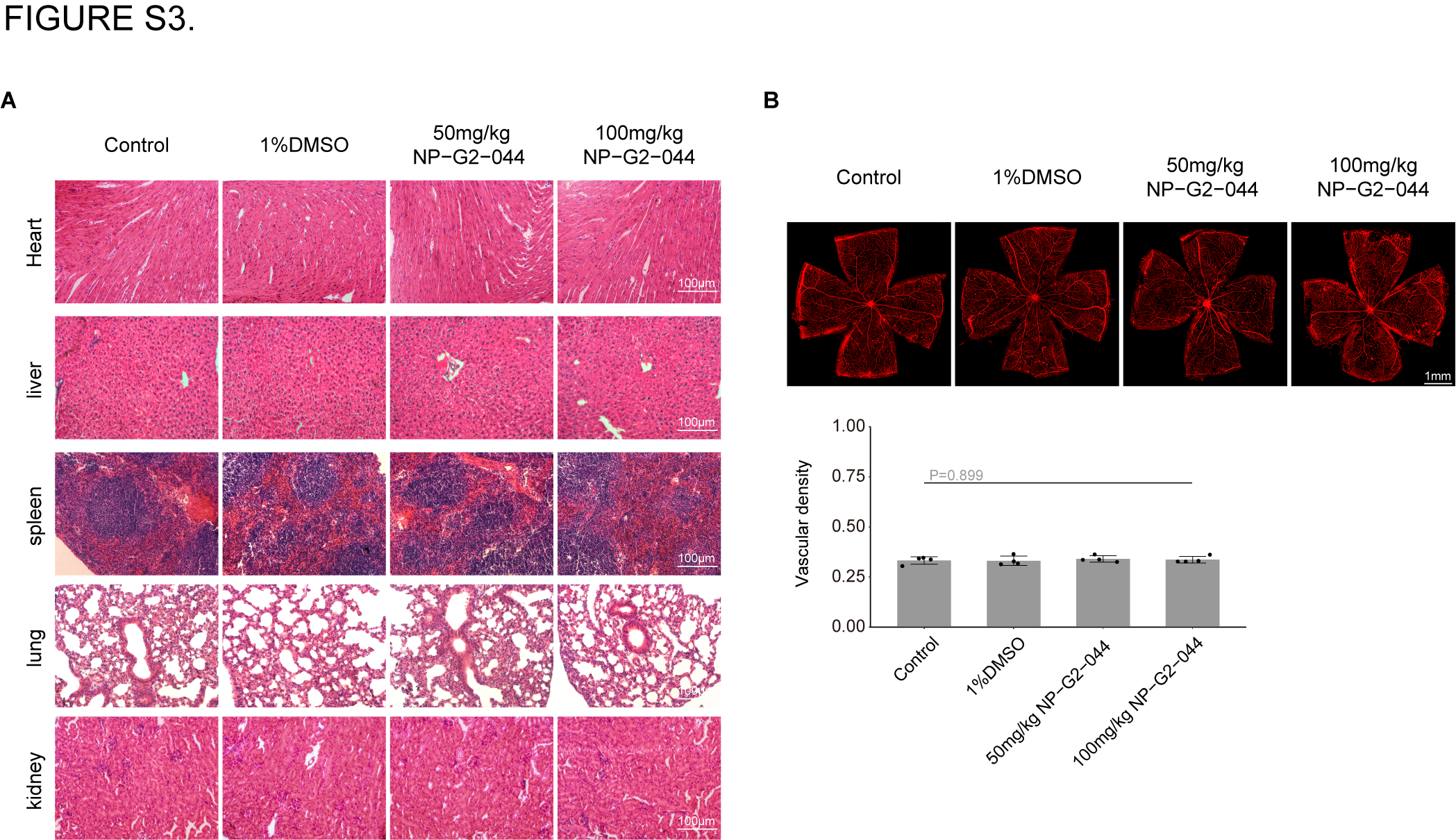

Supplement: Supplementary file 3 — Additional file 3: Figure S3. Vascular examination of mature retina and histological examination of heart, liver, spleen, lung, kidney. A IsoB4 staining of retinal flatmounts shows the vessel density in mice subjected to various treatments including PBS, 1%DMSO, 50 mg/kg NP-G2-044, or 100 mg/kg NP-G2-044 oral administration twice daily for 30 days. Scale bar:1mm. (n=4 independent experiments). Results are presented as mean ± SEM, statistical analyses were performed using One-way ANOVA with Bonferroni's post hoc test. (n = 4 mice per group). B H&E staining shows the morphology of heart, liver, spleen, lung and kidney in mice subjected to various treatments including PBS, 1%DMSO, 50 mg/kg NP-G2-044, or 100 mg/kg NP-G2-044 oral administration twice daily for 30 days. Oral administration of PBS is considered as the control group. Scale bar:100 μm. (n = 4 per group, data pooled from 4 independent experiments). [file 12967_2023_4225_MOESM3_ESM.tif]

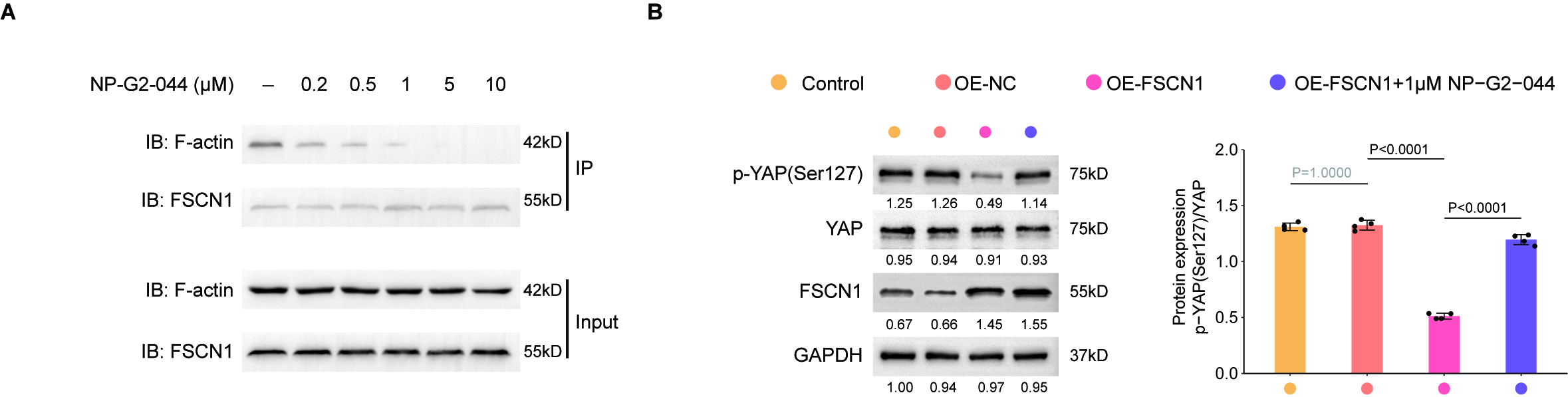

Supplement: Supplementary file 4 — Additional file 4: Figure S4. NP−G2−044 weakens the binding of FSCN1 to f-actin and inactivates YAP. A Coimmunoprecipitation (CO-IP) assays indicate the association of endogenous FSCN1 and F-actin in HRMEC cells after treatment with VEGF and different concentrations of NP−G2−044. (n=4 independent experiments). B Western blot assesses the protein expression of p−YAP(Ser127), YAP, FSCN1 and GAPDH in HRMECs transfected with OE-NC, OE-FSCN1, OE-FSCN1 added with 1μM NP−G2−044. Densitometric quantitation of Western blot band intensity shown in B. Results are presented as mean ± SEM, statistical analyses were performed using One-way ANOVA with Bonferroni's post hoc test. (n=4 independent experiments). [file 12967_2023_4225_MOESM4_ESM.tif]
