# Supplementary material for: Targeting FSCN1 with an oral small-molecule inhibitor for treating ocular neovascularization
Source: J Transl Med. 2023 Aug 18;21:555. doi: 10.1186/s12967-023-04225-0 (PMC10436462; doi:10.1186/s12967-023-04225-0)
Supplement: Supplementary file 5 — Additional file 5 : Additional materials and methods. [file 12967_2023_4225_MOESM5_ESM.docx]

**Cell culture and animals**

Human retinal microvascular endothelial cells (HRMECs) were purchased from ATCC and grown in serum-free high-glucose Dulbecco's modified Eagle medium (DMEM) (Gibco, C11995500BT) containing 10% FBS at 37 °C, 5% CO2 a­tmosphere. The primary mouse retinal and choroidal vascular endothelial cells (MRECs and MCECs) were isolated from tissues (see more below) and cultured in endothelial cell growth medium (ECM) [ScienCell,1001: FBS, Cat. No. 0025; endothelial cell growth supplement (ECGS, Cat. No. 1052); antibiotic (P/S, Cat. No. 0503)] as suggested by the supplier. The 20 ng/ml VEGF-A (Proteintech, 100-20) was used to create a pro-angiogenic culture condition and 200µM CoCl_2_ (Sigma-Aldrich, 232696) was used to create a hypoxic culture condition in vitro. All animals (C57BL/6J mice) were purchased from the Nanjing Qinglongshan Experimental Animal Center (Nanjing, China), handled in accordance with the ARVO Statement for the Use of Animals in Ophthalmic and Vision Research and approved by the Animal Care and Use Committee of Nanjing Medical University (Nanjing, China). All mice at different ages randomized in groups, including postnatal day 6 (P6) newborn mice, postnatal day 17 (P17) OIR mice, 6-week-old CNV mice and 12-month-old CNV mice, were fed standard chow diet and housed in a pathogen-free facility with no more than 5 mice per cage and were anesthetized in an isoflurane induction chamber and then sacrificed by cervical dislocation.

**Primary mouse retinal and choroidal vascular endothelial cells (MRECs and MCECs)**

Briefly, for qRT-PCR analysis of FSCN1 expression in endothelial cells of OIR model or CNV model, the retinas or choroids were digested into cell suspensions to extract primary retinal and choroidal vascular endothelial cells. For primary retinal vascular endothelial cells, retinas were isolated and cut into pieces as small as possible before enzymatic digestion in 1mg/ml collagenase I at 37 °C for 45 min; thereafter the digest was passed through a 45 µm cell strainer to filter the cell suspensions and remove undigested tissue fragments, centrifuged at 1000 rpm for 5 min, washed and resuspended with ECM to yield retinal cell suspensions. For primary choroidal vascular endothelial cells, RPE-choroid-sclera complexes were isolated and trypsinized with 0.05% trypsin (Thermo Fisher Scientific, 25300054) at 37°C for 15 min to detach RPE from the Bruch membrane with a small brush. Subsequently, choroid-sclera complex was minced into small pieces (2 x 2 mm^2^) and digested with 2 mg/ml collagenase II (BioFroxx, 2275MG100) containing 30 U/ml DNase I (Beyotime, D7073) in serum-free DMEM at 37°C for 60 min. Cell dissociation was by gentle pipetting up and down every 20 min. The digest was then triturated and filtered through a 70 µm cell strainer (Falcon, 352350), centrifuged at 1000 rpm for 5 min, washed and resuspended with ECM media to yield choroidal cell suspensions. Both retinal cellular suspensions and choroidal cellular suspensions were further purified using Dynabeads™ CD31 Endothelial Cell magnetic beads (Invitrogen, 11155D), and then cultured on 0.2% w/v gelatin-coated 6-well plates in ECM media for further qRT-PCR analysis.

**Oxygen-induced Retinopathy (OIR) Model**

Oxygen-induced retinopathy (OIR) model, a commonly recognized animal model for pathological retinal neovascularization, was established as previous described^47^ and the critical time was strictly applied as follows: (1) On postnatal day 7 (P7), neonatal C57BL/6J pups from the same litter were subjected to hyperoxic exposure (75% O2) for 5 days and nursing mums were replaced daily. (2) On postnatal day 12 (P12), pups were removed from hyperoxic environment to normoxic environment and pre-divided randomly into three groups on the basis of intravitreal injection of adeno-associated virus: control group, NC-ECKD group, and FSCN1-ECKD group. (3) On postnatal day 17 (P17) (maximum retinal neovascularization), pups were sacrificed and eyeballs were enucleated for preparation and staining of retina flatmounts.

**Laser-induced Choroidal Neovascularization (CNV) model**

Laser-induced Choroidal Neovascularization (CNV) model, a commonly recognized animal model for pathological choroidal neovascularization, was established as previous described^48^. In this study, the young (6-week-old) C57BL/6J mice were used for studies of pharmacodynamics and genophenotypes and aging (around 12-month-old) C57BL/6J mice were used for studies of anti-VEGF resistance. The eyes of anesthetized mice were treated with erythromycin eye ointment and covered with 10mm round Coverslips (Ted Pella, 260368), which were prepared and cleaned in advance with pre-moistened lens wipes (Zeiss). Four CNV lesions per eye were generated approximately 2 optic disc diameters from the optic nerve by laser photocoagulation (Quantel Medical; wavelength 577 nm), with a mode of slit-lamp, a spot size of 75 mum, a 100ms pulse duration and a 100mW incident power. The appearance of white and gaseous bubbles was seen at the time of laser photocoagulation, indicating the successful modeling with the rupture of Bruch's membrane. At 7 days after CNV induction (maximum choroidal neovascularization), mice were sacrificed and eyeballs were enucleated for preparation and staining of choroid flatmounts.

**Intravitreal injection**

Hamilton 10µl syringes (Hamilton Company, 7653-01) fitted with 34-gauge needles (Hamilton Company, 207434) were used for intravitreal injections. Under the aid of an operating microscope (Zeiss), a 40 mg/mL clinical concentration of Eylea (Bayer) was used for intravitreal injection. With the bevel of a 34G needle facing upward to carefully avoid blood vessels, an insertion slowly is made through the corneal limbus zone, and then tapping the syringe at the moment when the bevel is seen through the pupil. In CNV model, 1ul aflibercept stock solution was intravitreally injected on the bilateral eyes of each mouse following laser photocoagulation, under anesthesia.

**Retinal and choroidal flatmounts preparation**

Enucleated eyes were fixed in 4% paraformaldehyde (Sbjbio life Science, BL-G002) for 30 minutes at room temperature; thereafter a small incision was made at the limbus through a double-edged calibrated diamond knife (Fine Science Tools, 10318-14), followed by a circular-cut parallel to the limbus through a corneal scissor (Fine Science Tools, 15000-01). The anterior segment and lens were removed, and then the potential gap between neurosensory retina and RPE-choroid-sclera complex were exposed and insert by a corneal scissor to completely dissociate retina and choroid. Four radial relaxing incisions were made through a diamond knife, so that the retina and choroid could be flattened. For retinas from postnatal mice, particular attention needs to be paid to clean residual hyaloid vascular system, avoiding coverage of superficial retinal vessels. For choroids from CNV mice, particular attention needs to be paid to observe the position of the laser spots, avoiding disruption of CNV spots.

**Isolectin B4 staining and quantitative analysis**

To detect vascular system, retinal and choroidal flatmounts were permeabilized in 5% bovine serum albumin with 1% Triton X-100 for 45min at 37℃ and then incubated with 10ul biotinylated Griffonia simplicifolia isolectin B4 (IsoB4) (Thermo Fisher Scientific, I21413, 1:50) per flatmount overnight at 4℃. After rinsing in PBS, the flatmounts were photographed under an inverted microscope (10x magnification) (Olympus Corp, IX73P1F). Then, the photo stitching was done in Adobe Photoshop CS6 to show the image of the whole retina and choroid. By Fiji (ImageJ) software, quantification and analysis was performed as follow: For retinas from postnatal day 6 (P6) newborn mice, vascular density was determined by the number of pixels of isolectin B4 signal normalized to total retinal area; Radial length was determined by the longest distance from optic disc to vascular front; Number of tip cells or filopodia was determined using Cell Counter plugin; Length of filopodia was measured from the cell body of tip cells to the boundary of filopodia using the segmented line tool. For retinas from postnatal day 17 (P17) OIR mice, Photoshop was used to fill the neovascular tufts with white color and fill the avascular area with red color manually, then the area was measured using Color Threshold function. For choroids from CNV mice, CNV area was determined and converted from pixel number to physical dimensions (μm^2^).

**Immunofluorescence labeling and colocalization Analysis**

Retinal and choroidal flatmounts were fluorescently labeled FSCN1 to identify the precise location between FSCN1 and vascular endothelial cells. After Isolectin B4 staining, whole-mount immunofluorescence labeling was performed using anti-FSCN1 primary antibody (Abcam, ab126772, IF 1:200) followed by an Alexa 488-conjugated secondary antibody (Abcam, ab150077, IF 1:400). The quantitative colocalization analysis of FSCN1 fluorescence (green channel) and IsoB4 fluorescence (red channel) was performed with Fiji (ImageJ) JACoP plugin to calculate Manders' coefficient.

**Transwell assay**

4 × 10^4^ HRMECs were seeded into upper chambers of 24-well transwell inserts (8 µm PET, 24-well Millicell) containing 200 µl DMEM media supplemented with 0.1% FBS. Meanwhile, the lower chambers were filled with 600µl DMEM supplemented with 10% FBS to induce cells through polycarbonate membranes (8 µm pore size) into high nutrient environment. After migration for 16 hours, cells on the undersurface of the insert were fixed in 100% methanol solution for 15 minutes at room temperature, followed by stained with 0.05% crystal violet solution (Sigma-Aldrich, 548-62-9) in PBS for 40 minutes. Prior to photograph taken on under an inverted microscope, cells on the upper surface of the inserts were gently scrubbed with PBS-saturated cotton swabs. The number of migrated cells was determined using Fiji (ImageJ) Cell Counter plugin.

**Wound scratching assay**

Briefly, HRMECs were plated into 6 well plate and cultured until 90% confluency. With the aid of a ruler, the cells layer was scratched vertically by a sterile 10µl pipette tip (Thermo Scientific, 9400303) to generate an approximately 1mm wide wound. After 24 hours, DMEM media was removed and alternative by PBS to clean dead cells. Subsequently, HRMECs were stained with rhodamine-conjugated phalloidin (Abcam, ab235138, 1:1000) and DAPI (Abcam, ab228549, 1:1000) to visualize the actin cytoskeleton and nuclei, respectively. The wound area was determined using Fiji (ImageJ) polygon selection function.

**Tube formation assay**

24-well plates, pipette tips and BD Matrigel Matrix (Corning,356234) were placed at 4℃overnight before use. Pre-thawed 35µl BD Matrigel Matrix was added into a 24-well plate per well using ice-cooled pipette tips, and then polymerized at 37℃ for 30 min. Subsequently, resuspended HRMECs (5 x 10^4^ cells) were seeded on polymerized BD Matrigel Matrix and cultured in 1ml DMEM supplemented with 10% FBS for 5 hrs to allow for formation of vascular tube-like structures. The tube formation length was determined using Fiji (ImageJ) Angiogenesis Analyzer plugin.

**Three-dimensional (3D) bead sprouting assay**

Three-dimensional bead sprouting assay was performed as follow: On day 0, 0.1 g dry Cytodex@ 3 microcarrier beads (Sigma-Aldrich, C3275-10G) were hydrated in a 10 ml PBS to reach approximately 30,000 beads/ml. Fibrinogen solution was prepared from 20mg of fibrinogen (Sigma-Aldrich, F8630-1G) dissolved in 10 ml of DPBS (Thermo Fisher Scientific,14190144). Aprotinin stock solution was prepared from 10mg of aprotinin (Sigma-Aldrich, A1153) dissolved in 8 ml of deionized water (4 U/ml). Thrombin stock solution was prepared from 1000 Units of thrombin (Sigma-Aldrich, T4648-1KU) dissolved in 20 ml of deionized water (50 U/ml). On day 1, HRMECs were cultured to 100% confluence in 24 well plate, and cells of 4 wells (approximately 1.6 x 10^6^ cells) were trypsinized and resuspended in 1ml EGM-2 media and then mixed with 120 ul beads solution (approximately 3600 beads) in 2 ml EGM-2 media. A total of 3ml HRMECs/beads suspension were gently mixed by inverting the tube every 20 min for 4 hours at 37 ℃ and cultured in a T-25 flask overnight. On day 2, beads coated with HRMECs were transferred to a 10ml conical tube, washed twice in 1ml EGM-2 media, dispersed in a fibrinogen/aprotinin mixture (6ml fibrinogen solution plus 225µl aprotinin stock solution), and evenly added to 12 wells of a 24-well plate pre-coated with 300µl thrombin stock solution. The plate was not disturbed so as to solidify for 5 min at room temperature and then incubated at 37 °C for 30 min. While waiting, HFSF cells were trypsinized and resuspended in 1ml EGM-2 media and then 20ul cellular suspension (approximately 2 x 10^4^ cells) with 1ml EGM-2 media was added to the center of the clot per well. EGM-2 media was replaced every other day (day 4, 6). On day 7, images were captured under an inverted microscope (Olympus Corp, IX73P1F). The number of sprouts per bead and the average length of sprouts were determined using Fiji (ImageJ) Sprout Morphology plugin.

**Rhodamine-phalloidin staining**

For morphological analysis of HRMECs, Rhodamine-phalloidin staining was performed to visualize the actin cytoskeleton and filopodia. HRMECs were fixed in 4% paraformaldehyde (PFA) for 20 minutes at room temperature, followed by incubation with Rhodamine conjugated phalloidin (Abcam, ab235138,1:1000) for 30min at room temperature. The number and average length of filopodia per cell were determined using Fiji (ImageJ) FiloQuant plugin.

**Cell apoptosis assay**

Apoptosis was determined by FACS flow cytometry using Annexin V-FITC/PI Apoptosis Detectiion Kit (Vazyme, R323-01). Briefly, the cells collected after trypsinization were washed with PBS and resuspended with binding buffer. Cells were then stained with 5 μl Annexin V-FITC and 5 μl of PI Staining Solution according to the manufacturer’s instructions. The cells were acquired by the flow cytometer (Beckman Coulter lnc, A00-1-1102) and analyzed with FlowJo 10.4 software (Tree Star, Ashland, OR, USA).

**MTT assay**

Cytotoxicity was evaluated by 3-(4,5-dimethylthiazol-2-yl)-2,5-diphenyltetrazolium bromide (MTT) assay in seven cell lines, including HRMEC, MREC, MCEC cells. Briefly, 4 × 104 cells were seeded in a 96-well plate with repeated 6 wells per groups, cultured overnight, and then treated with different concentrations of NP-G2-044 for 48hr. After the supernatant was removed, 30µl 5mg/mL MTT (Invitrogen, M6494) in phosphate buffer solution (PBS) was added to each well and incubated for 3h. Subsequently, MTT solution was removed and the formed formazan was extracted and dissolved in acidified isopropanol solution, and the optical density at 595 nm was measured with a microplate reader (Molecular Devices lnc, 26 270-1189).

**Tissue paraffin section and staining (H&E and tunnel staining)**

For paraffin embedding, eye cups without lenses were fixed in 4% paraformaldehyde (PFA) overnight at 4 ℃, dehydrated through ethanol gradient (50%, 60%, 70%, 80%, 90%, 100%), made transparent in xylene, and embedded in paraffin wax. Paraffin-embedded 5 mm sections covering entire retina were made along the vertical meridian of the globe, mounted on glass slides, and then stored at room temperature. The retinal cross-sections were stained with a hematoxylin and eosin (HE) staining kit (Beyotime, C0105) to assess histopathological changes, and a tunel staining kit (Beyotime, C1098) was used to evaluate apoptosis in tissues, respectively, according to the manufacture's instruction.

**Visual electrophysiology (ERG) testing**

In brief, mice were dark-adapted overnight (at least 8 hrs), and then anaesthetization and mydriasis were conducted under dim red illumination (720 nm), followed by ERG recordings on a 37 ℃ heating pad in complete darkness. ERG responses were recorded by placing a main active electrode on the corneal apex with ear subcutaneously and a ground electrode on the tail subcutaneously. With the responses to 50 ms short-wavelength flashes of a 2.0 cd s/m2 flash stimulus by the photopic stimulator (Tomey EP-1000 device, Nagoya, Japan), ERG traces of retinal irradiance were acquired with a band-pass filtered between 1Hz to 300Hz.

**Visual cliff test**

Visual ability was assessed by visual cliff test. The test relies on the visual depth perception of animals to avoid the deep side of the field. The home-made visual cliff apparatus was created as follows: Two transparent acrylic boxes (40 x 20 x50 cm) were assembled side by side to form a square platform region, with a long wooden slat (40 x 4 x 2 cm) placed on the junction, so as to divide the platform region into two halves designated the safe and cliff side. To give the visual illusion of a cliff, the safe side of platform region and the cliff side of floor were patterned with black and white grids (2.5 x 2.5 cm squares). All mice were placed onto the center wooden slat and their choice of the side from which to descend were recorded. Each mouse was subjected to the test ten times, and the apparatus were thoroughly cleaned by 75% disinfectant alcohol after each test.

**Light/dark transition test**

Visual ability was additionally assessed by light/dark transition test. The test relies on the innate tendency of animals to prefer dark environment rather than light environment. The light/dark box apparatus consists of a lighted compartment (two-third, 200 lux) and a dark compartment (one-third, 0 lux). All mice were dark adapted for 12 h and then allowed to freely explore the whole apparatus in the dark environment for 10 min before each test. To initiate testing, mice were placed into the lighted compartment facing the dark compartment and the total time spent in the dark compartment were recorded. Each mouse was subjected to the test once, and the apparatus were thoroughly cleaned to eliminate traces of mice smell after each test.

**Co-Immunoprecipitation (Co-IP)**

To obtain whole cell extract containing stabilized F-actin, we lysed HRMEC cells at 4℃ with actin stabilizing buffer (1% Triton-X 100, 10 mM EDTA, 0.1% SDS, 1% sodium deoxycholate, 50 mM NaCl, 5 mM MgCl2,1 mM ATP and 10 mM Tris-HCl, pH 7.5) supplemented with a protease inhibitor mixture (Roche, 11697498001). After centrifugation at 12000 rpm for 30min, the supernatant fraction was harvested as the total cellular protein extract and then incubated with SureBeads (BioRad, 161-4833) previously coated with anti-FSCN1 antibody (Abcam, ab126772) or control IgG antibody (Abcam, ab172730) at room temperature for 1 hour. FSCN1- bound proteins complexes were eluted from the beads by boiling in 2x SDS-PAGE sample loading buffer for 10 min (Beyotime, P0288), and analyzed by Western blot as described above.

**Active CDC42 assay**

Relative levels of GTP‐bound Cdc42 (Cdc42-GTP) were determined by Cdc42 Activation Assay Kit (Abcam, ab211163). Briefly, HRMEC (2 × 106) were lysed in RIPA lysis buffer (Beyotime, P0013B) supplemented with a protease inhibitor mixture (Roche, 11697498001). Samples were incubated with PAK‐1 PBD agarose beads and bound (activated) as well as unbound (non‐activated) Cdc42 fractions were probed by immunoblotting with an anti‐Cdc42 antibody (BD Biosciences, 610929, 1:500). Activated protein was normalized to total protein and β‐actin, and the relative amount was quantified as Western Blot.

**Nuclear/Cytoplasmic protein fractionation**

The fractionation of cytoplasm and nucleus were separated with a subcellular protein fractionation kit (Thermo Scientific, 87790). For cytoplasmic protein fractionation, cells were lysed with cytoplasmic extraction buffer (CEB) for 30 min at 4 °C and the supernatant (cytoplasmic extract) was collected by centrifugation at 13,200 rpm for 30 min. For nuclear protein fractionation, the centrifugal precipitate was suspended and incubated in nuclear extraction buffer (NEB) for 30 min at 4 °C and the supernatant (nuclear extract) was collected by centrifugation at 13,200 rpm for 30 min. In this study, the HRMEC cells were harvested to isolate the cytoplasm and nucleus, and cytoplasmic and nuclear expression of YAP were analyzed by immunoblotting to determine YAP nucleocytoplasmic shuttling.
